# Influence of Hypoxia on Tumor Heterogeneity, DNA Repair, and Cancer Therapy: From Molecular Insights to Therapeutic Strategies

**DOI:** 10.3390/cells14141057

**Published:** 2025-07-10

**Authors:** Dominika Kunachowicz, Paulina Tomecka, Mikołaj Sędzik, Jarosław Kalinin, Jacek Kuźnicki, Nina Rembiałkowska

**Affiliations:** 1 Department of Pharmaceutical Biochemistry, Faculty of Pharmacy, Wroclaw Medical University, Borowska 211a, 50-556 Wroclaw, Poland; dominika.kunachowicz@student.umw.edu.pl; 2 Faculty of Medicine, Wroclaw Medical University, 50-367 Wroclaw, Poland; p.tomecka@student.umw.edu.pl (P.T.); mikolaj.sedzik@student.umw.edu.pl (M.S.); jaroslaw.kalinin@student.umw.edu.pl (J.K.); jacek.kuznicki@student.umw.edu.pl (J.K.); 3 Department of Molecular and Cellular Biology, Faculty of Pharmacy, Wroclaw Medical University, Borowska 211a, 50-556 Wroclaw, Poland

**Keywords:** hypoxia, cancer, tumor heterogeneity, DNA repair, cancer therapy, therapeutic resistance, hypoxia-inducible factors (HIFs), tumor microenvironment, hypoxia-targeted therapy

## Abstract

Hypoxia, characterized by a reduction in tissue oxygen levels, is a hallmark of many solid tumors and affects a range of cellular processes, including DNA repair. In low-oxygen conditions, cancer cells often suppress key DNA repair pathways such as homologous recombination (HR), leading to the accumulation of DNA damage and increased genomic instability. These changes not only drive tumor progression but also contribute to resistance against conventional therapies. Hypoxia significantly reduces the effectiveness of oxygen-dependent treatments, including radiotherapy and many chemotherapeutic agents. To address this limitation, bioreductive drugs have been developed that become selectively activated in hypoxic environments, providing targeted cytotoxic effects within oxygen-deprived tumor regions. Additionally, the rapid growth of tumors often results in disorganized and inefficient vasculature, further impairing the delivery of oxygen and therapeutic agents. This review explores the molecular mechanisms by which hypoxia disrupts DNA repair and contributes to treatment resistance. It also presents emerging therapeutic strategies aimed at targeting the hypoxic tumor microenvironment to improve treatment efficacy and patient outcomes.

## 1. Introduction

Hypoxia is a pathological condition characterized by reduced oxygen availability in tissues, leading to impaired cellular function [1]. In normal tissues, oxygen is essential for maintaining cellular homeostasis, supporting metabolic activity, and ensuring efficient DNA repair. However, in tumors, the rapid proliferation of cancer cells outpaces oxygen supply, creating a hypoxic microenvironment that drives disease progression. It is a defining feature of tumors, occurring in 90% of solid malignancies [2]. Hypoxia is associated with poor prognosis in cancers such as prostate, cervical, and breast cancer [3]. According to studies, PO_2_ in breast tumors ranges from 2.5 to 28 mmHg (median 10 mmHg), compared to 65 mmHg in healthy tissue [4]. Oxygen levels within hypoxic tumor regions fluctuate between 1–2%, with some areas experiencing severe hypoxia (0.2%) or anoxia (0%), depending on tumor size, stage, and proximity to microvessels [5]. This variability in oxygen distribution contributes to tumor heterogeneity, influencing cellular adaptation, genetic instability, and therapy resistance.

In normal cells, hypoxia typically results in cell death; paradoxically, it can drive genomic changes that enable tumor cells to adapt to nutrient deprivation and a hostile microenvironment, promoting their survival [6]. Tumor hypoxia induces multiple cellular adaptations, including altered gene expression, suppression of apoptosis, activation of autophagy, promotion of epithelial–mesenchymal transition (EMT), malignant progression, metastasis, and metabolic reprogramming [7]. Oxygen deprivation or hypoxia leads to oxidative stress, triggering the production of hypoxia-inducible factors (HIF) and reactive oxygen species (ROS) [8]. Numerous genes have been recognized as HIF-1 targets, including GLUT-1, GAPDH, and VEGF, which play roles in energy metabolism, cell survival, and angiogenesis [9]. These adaptations support tumor survival by reducing oxygen dependency, shifting metabolism from oxidative phosphorylation to glycolysis, and lowering energy demands for essential cellular processes such as proliferation [10].

**Figure 1:**
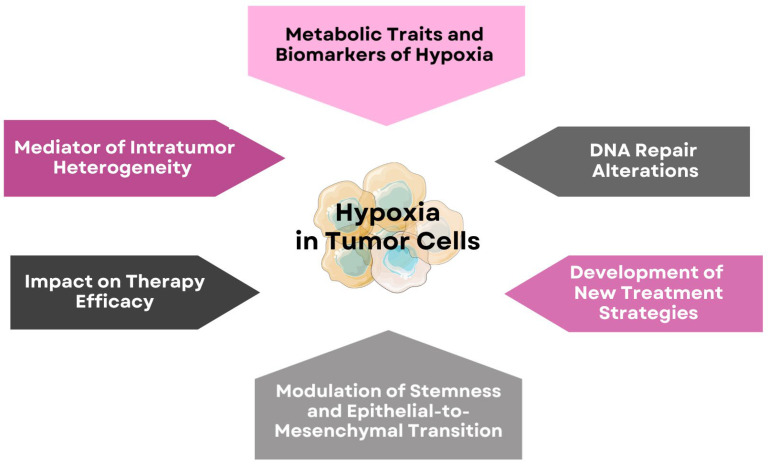
Key Biological Consequences of Hypoxia in Tumor Cells.

Tumor cells exhibit distinct phenotypic states based on oxygen distribution. Cells located near functional blood vessels in well-oxygenated regions remain viable and proliferative [11]. As the distance from blood vessels increases, oxygen levels drop, and at approximately 150 μm away, cells may become anoxic, leading to necrotic patches. Some cells adapt to hypoxic conditions and can survive in oxygen levels as low as 1% PO_2_. Interestingly, these hypoxia-adapted cells may later migrate back to oxygen-rich regions, demonstrating the plasticity of tumor cell behavior. Meanwhile, nonviable cells accumulate within the necrotic core [6]. This dynamic distribution of tumor cells based on oxygen levels highlights the complex interplay between hypoxia, tumor architecture, and cellular adaptation mechanisms. As illustrated in Figure 1, hypoxia influences multiple aspects of tumor biology, including metabolic adaptation, therapy resistance, and DNA repair alterations.

Hypoxia enhances tumor aggressiveness by driving clonal selection, favoring highly invasive cancer cell populations. These adapted clones further exacerbate hypoxia, creating a self-perpetuating cycle that diminishes the effectiveness of standard therapies such as radiotherapy, chemotherapy, and phototherapy [12]. Importantly, well-oxygenated cells are significantly more susceptible to ionizing radiation than hypoxic cells, as oxygen enhances DNA damage and prevents repair. Consequently, tumor hypoxia is a major factor in predicting the response to chemotherapy and radiotherapy [13].

A critical mechanism by which hypoxia contributes to therapy resistance is through its impact on DNA repair. Hypoxic tumor regions promote genomic instability by disrupting DNA repair pathways, increasing resistance to treatment [2]. Hypoxia specifically suppresses homologous recombination (HR), an accurate DNA repair mechanism, while promoting non-homologous end joining (NHEJ), a more error-prone pathway. This shift favors genomic instability and enables cancer cells to withstand DNA-damaging therapies, contributing to treatment resistance and tumor progression [14].

Tumor hypoxia exhibits significant spatial and temporal heterogeneity. Acute hypoxia arises within minutes to hours due to transient perfusion disruptions, while chronic hypoxia, resulting from oxygen diffusion limitations, persists for hours to days [15]. Acute hypoxia contributes to genomic instability by delaying DNA damage responses and triggering p53-dependent apoptosis. Notably, cells lacking functional p53 are particularly prone to genetic instability and tumorigenesis upon reoxygenation following acute hypoxic exposure. Chronic hypoxia, on the other hand, leads to persistent suppression of DNA repair mechanisms, including homologous recombination and mismatch repair, promoting replication errors, double-strand breaks, and mutagenesis [16].

**Figure 2:**
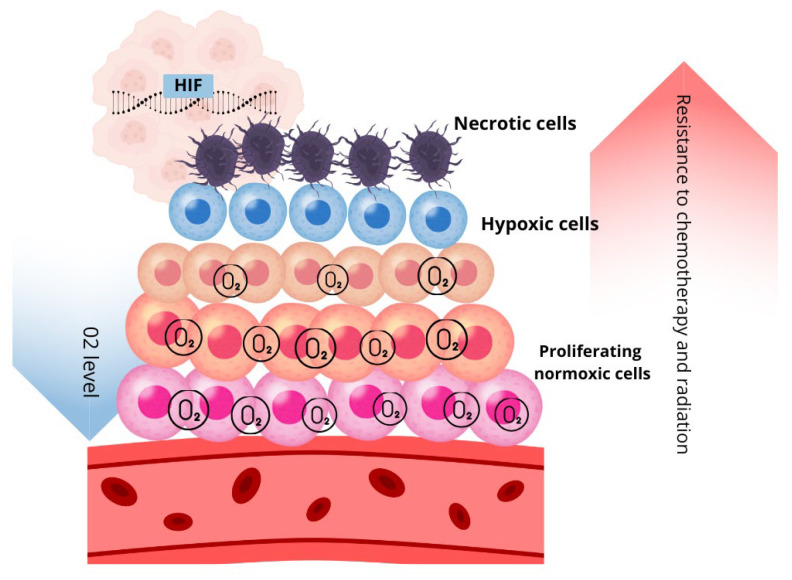
The link between tumor oxygen levels and therapy resistance. Oxygen decreases with distance from blood vessels, leading to hypoxia and necrosis. Hypoxia-inducible factors (HIFs) drive adaptations that enhance survival. As hypoxia increases, resistance to chemotherapy and radiation rises, making treatment less effective.

Given the profound impact of hypoxia on DNA repair, genomic instability, and therapy resistance, addressing hypoxia is a crucial strategy for improving cancer treatment. Understanding the complex interplay between hypoxia, heterogeneity, DNA repair mechanisms, and therapy resistance is essential for improving cancer treatment outcomes. This review will analyze the molecular mechanisms through which hypoxia modulates DNA repair pathways, contributing to resistance to radiotherapy and chemotherapy. Furthermore, it will evaluate therapeutic strategies targeting the hypoxic tumor microenvironment to enhance treatment efficacy. Figure 2 illustrates how these oxygen-driven dynamics contribute to therapy resistance.

## 2. The Interrelationship Between Tumor Heterogeneity and Hypoxia

### 2.1. The Concept of Tumor Heterogeneity: Origins and Effects

A large body of evidence gathered throughout the recent decades of research has proven that cells composing cancerous lesions are not uniform in nature, but evolve in time under selective pressures, which promote the formation of diversified cell populations. There are several levels of such diversification, including genetic, epigenetic, phenotypic, and functional alterations between various sets of cancer cells, known as intratumoral heterogeneity (ITH). This phenomenon is known to strongly mediate drug resistance development, acquisition of stem cell-like properties, and metastatic ability of cancer cells, dictating their interactions with each other and with non-malignant components of TME [17,18].

According to the generally acknowledged concept, cancer originates from a cell that has accumulated somatic driver mutations leading to its reprogramming and malignant transformation toward unlimited proliferation [19]. Such a cell gives rise to the mutant clones, accumulating further genetic and, in consequence, phenotypic and functional aberrations. The proliferation of such cells further leads to diversification into multiple subclones, among which those exhibiting growth and survival advantage are favored, allowing for tumor expansion. Thereby, ITH is a major factor underlying therapy failures and cancer recurrence due to the selective outgrowth of the resistant cell populations [20].

Several groups of factors underlying ITH, such as genetic, epigenetic, and microenvironmental, can be listed; however, they shall not be considered separately since their interrelationship contributes to the whole complicated picture. Genetic heterogeneity remains best studied; originally, the broadest knowledge was related to small-scale point mutations such as single-nucleotide variants due to ease of studying, but currently, with the development of increasingly advanced and sophisticated research tools and techniques, deeper insight into rearrangements on higher DNA organizational levels is being gained. What can be expected is that these large-scale modifications have a greater impact on cell function and phenotype [21]. In cancer cells, variations in chromosome structure and number due to their incorrect segregation during mitosis, such as aneuploidy (changes in the number of chromosome copies), whole-genome duplications, and chromoplexy (presence of multiple chained translocations) are common, comprising up to 90% of cancer genomes. These are representations of chromosomal instability, which is often understood as a consequence of mutations in genes involved in various stages of cell division, defective DNA damage repair, and errors in mitotic spindle assembly. As a result, an increased rate of structural and numerical changes in chromosomes leads to an increase in genetic variations among cells composing the whole tumor population, endowing some of them with greater capacity to adapt to the challenging microenvironment, under hypoxic conditions, and where nutrient supply is limited. Moreover, genomic instability was proved to promote heterozygosity loss in tumor suppressor genes, which is a step toward their inactivation [21,22].

Tumor heterogeneity is also expressed in epigenetic traits; however, to date, it remains poorly characterized. Epigenetics, understood as control over gene activity unrelated to changing DNA sequence, operates through DNA methylation, histone modifications, and non-coding RNAs. Unlike genetic alterations, epigenetic changes are reversible [23]. DNA methylation patterns remain the most studied due to the quantitative nature and relative ease of measurement. For example, heterogeneous methylation in different clones was observed for some cases of melanoma and primary endometrial cancer. In certain genome-wide sequencing-based studies, metastatic melanoma cells were characterized by global hypomethylation in comparison to their matched primary cells [24]. Similarly, single-cell-level analysis of colon cancer clonal populations has shown that methylation loss is correlated with mesenchymal-like transcriptional identity [25]. However, no clear conclusions can be established based on the existing data. Lung adenocarcinoma tissues, as reported by Dietz et al., presented a high level of heterogeneity within a single sample; methylation profiles differed between each other to the extent that the samples would originate from different individual tumors [26].

The tumor microenvironment (TME), consisting of matrix proteins, glycoproteins, and glycans along with non-cancerous stromal, immune, and endothelial cells, is a dynamic ecosystem that supports cancer progression. Cancer-associated fibroblasts (CAFs), the most abundant cell population in the TME, provide cancer cell growth factors, cytokines, and chemokines for proliferation and survival, and their ability to modify the composition and structure of ECM facilitates tumor metastasis [27]. Along with immune cells, such as T cells, B cells, and macrophages, CAFs can exhibit various functional phenotypes and spatiotemporal distribution within TME, supporting the phenomenon of microenvironmental heterogeneity [28,29]. Moreover, the network of TME evolves together with the progression of cancer, shaped by changing genetic and epigenetic changes occurring in proliferating precursor cells and then malignant cells during clonal selection. Thereby, variability in composition and proportion of non-neoplastic cells, structural components, and soluble factors in the TME is connected with functional and phenotypic alterations of tumor cells [30]. Apart from being subjected to heterogeneity, as described above, diverse TME components can influence phenotypic heterogeneity of cancer cells through complex, multi-directional interactions, pointing toward their interdependence [31].

Differential genetic, epigenetic, and microenvironmental influences are reflected in cell phenotypic and metabolic heterogeneity. The most significant feature of cancer cell metabolism, altered in comparison to normal cells, is a switch in energy acquisition from oxidative phosphorylation to glycolysis, first described by Otto Warburg. Utilization of the glycolytic pathway is, in large part, promoted by HIF-1 and is a mechanism that allows cancer cells to maintain high proliferative ability in conditions of reduced oxygen availability [32]. Although less effective in generating ATP, glycolysis provides precursors to produce macrobiomolecules, such as proteins or lipids, for producing reducing equivalents essential to perform cell division. Often, these cells use metabolites (e.g., glutamine, ketones, or lactate) from external sources, such as cells comprising the tumor microenvironment, to build their own structures and increase mass. Thereby, glycolytic cancer cells are characterized by more rapid proliferation [33]. As can be expected, cancer cells differ from each other in their metabolic phenotypes, which is supported by the results of metabolomic studies and metabolic flux analyses at the single-cell level. Different metabolic subtypes can arise upon the influence of metabolism-related genetic factors; for instance, phosphoglycerate dehydrogenase-encoding gene amplification in certain, but not all, cancer cells leads to an increase in serine synthesis. The expression of numerous genes controlling metabolic processes can also be altered by epigenetic factors. As an example, glycolytic flux-enhancing hexokinase 2 (HK2) gene promoter hypomethylation can be mentioned. Interactions between cancer cells and their ECM, nutrient availability, and oxygenation level, which will be further discussed, are also important heterogeneity-driving factors [34,35].

### 2.2. Hypoxia as Mediator of Intratumoral Heterogeneity

#### 2.2.1. Spatial and Temporal Distribution of Oxygen

The spatial oxygen distribution within tumors is also not uniform. Also, the exact values of oxygenation depend on the size, stage, and initial oxygen level in the specific tumor setting and architecture. For instance, in head and neck cancers, a hypoxic core and normoxic boundaries are present, while in breast cancers, this pattern is not visible due to their multinodular structure with multiple zones of both necrotic and proliferating cells [36].

According to common understanding, oxygen-rich (normoxic) regions are typically located near capillaries, while increasing distance from the vasculature results in a hypoxia gradient, ranging from mild (around 3% O_2_), through severe (often below 0.02% O_2_), to anoxic conditions (<0.001% O_2_). In consequence, regions located deeply inside the tumor, deprived of vasculature and, thereby, proper oxygen supply due to limited diffusion, would be characterized by severe hypoxia and the occurrence of necrotic sites. However, this is true only for regular, functional blood vessels. Cancer vasculature is usually corrupted and disorganized, with numerous spatial differences in vessel density and the level of perfusion. Thereby, the flow of oxygen is alternating, exposing surrounding cells to perfusion fluctuations. It leads to scenarios in which cells comprising a tumor are exposed to diverse combinations of hypoxia severity and duration, triggering differential cellular responses [5,37,38].

In light of the above, hypoxia should not be regarded as a static condition; however, the term chronic hypoxia is used to describe a prolonged state in which specific tumor regions are persistently exposed to low oxygen levels. In contrast, exposure to its variable and fluctuating concentrations leads to dynamic oxygenation and states of cyclic (intermittent) hypoxia, often characterized by changes between deep and moderate hypoxia and, what is even more explicit, events of hypoxia and rapid rises in oxygen levels. It has been shown that cycling hypoxia has a more pronounced positive effect on cancer cell survival, metastasis, and chemoresistance than the chronic variant [39,40]. It is mainly due to the reoxygenation phases, which, after hypoxic periods, have profound effects on cancer cells through oxidative damage mediated by abrupt ROS generation. This leads to an increased mutation rate and errors in the DNA repair system, thereby fueling tumor heterogeneity and promoting the selection of the most resilient cells with enhanced survival, metastatic capacity, and therapy resistance. Moreover, cycling hypoxia contributes to cancer progression by facilitating new blood vessel formation, intensifying tumor-promoting inflammation, and activating survival-related pathways in cancer cells [39,41]. Knowing their involvement in modulating cellular metabolism, ROS seems to have a remarkable contribution to tumor heterogeneity; however, there are limited studies evaluating this topic [42].

#### 2.2.2. Hypoxia and Metabolic Heterogeneity

Hypoxia is one of the major drivers of metabolic heterogeneity in cancer cells. One of the ways to increase the survival of cancer cells under hypoxic stress is by inducing a metabolic switch from oxidative phosphorylation to glycolysis. Due to enhanced glycolysis, the cytosol becomes acidotic as the accumulating pyruvate is not oxidized, but converted into lactate. The excess lactate is eliminated from the cell by upregulating monocarboxylate transporters, acidifying the surrounding microenvironment, which is a hallmark of cancer phenotype, owing to promoting metastatic ability. The more severe the hypoxia, the higher the glycolysis rate and, thereby, lactate production than the intensity of the OXPHOS and tricarboxylic acid (TCA) cycle [33,43]. HIF-1α is a powerful glycolysis-inducing factor through binding to hypoxia-responsive elements, which are present in the promoters of genes encoding key glycolytic enzymes and transporters, such as glucose transporters, hexokinase 2, lactate dehydrogenase A, and monocarboxylate transporters [44]. Interestingly, a kind of symbiosis can be developed between populations of cells located in differently oxygenated tumor sites: while hypoxic cells predominantly produce lactate via glycolysis and export it through monocarboxylate transporter 4, cells lying in sites richer in oxygen uptake lactate and utilize it in OXPHOS as the energy source [45].

A spectrum of changes in mitochondrial redox status, mediated by decreased availability of O_2_, can also be noticed. Conditions of mild hypoxia increase ROS production, primarily through NADPH oxidase activation and subsequent superoxide generation. In contrast, chronic or severe hypoxia often leads to adaptive responses that reduce ROS levels through p53-mediated antioxidant defense activation. However, there are many unknowns concerning the exact mechanisms of ROS implication in tumor heterogeneity [42,46]. Other effects of decreased O_2_ tension, at levels attributable to severe hypoxia, include lowering the rate of fatty acid desaturation, cholesterol synthesis, and an increase in lipid uptake [43]. The interconnection between epigenetic alterations and cellular metabolism is also worth mentioning; hence, varying oxygenation can influence metabolic heterogeneity indirectly [6].

Since hypoxia influences epigenomic and transcriptomic profiles, hypoxic and normoxic cancer cells present different potentials in terms of proliferative, metastatic, apoptotic, and angiogenic capacity, overall contributing to global tumor heterogeneity [36]. For example, it has been shown that histone-modifying enzyme expression and activity can be modified by hypoxia, like histone methyltransferase G9a stabilization observed in such conditions. Also, HIFs’ implication in regulating transcription of various factors engaged in chromatin remodeling, and, thereby, global chromatin alterations. Moreover, hypoxia is partly responsible for DNA hypermethylation in tumor suppressor genes by deactivating ten–eleven translocation enzymes, contributing to the heterogeneity of cancer cells [47].

#### 2.2.3. Hypoxia and Different Cancer Cell Phenotypes

- Cancer Stem Cells

A hypoxic environment has been found to largely mediate the development and maintenance of cancer cells’ phenotypic diversity. Longstanding research allowed us to conclude that hypoxia contributes to disease progression by promoting cancer stem-like, metastatic, and chemoresistant phenotypes [17]. A population of cancer stem cells (CSCs), capable of self-renewal, is known as the major villain of cancer progression, metastasis, and recurrence due to their unique characteristics like high plasticity and extended periods of quiescence, leading to enhanced survival and resistance to treatment targeting rapidly proliferating cells [12]. CSCs have been noticed to settle within the hypoxic regions of the tumor, and the following studies have proven that indeed such conditions preserve stemness, while an increase in oxygen levels leads to the opposite effect [48]. This is attributable to certain molecular events, initiated by master hypoxia regulators, which are HIFs. Although both HIFs are stabilized under hypoxia, HIF-2α is often more selectively expressed in CSC populations and has been shown to promote self-renewal and the expression of stemness-associated genes more robustly than HIF-1α. Moreover, the response mediated by HIF-2α is related to chronic hypoxia and activation of Oct4 through the direct interaction with its promoter, c-Myc, and SOX2, while HIF-1α mainly triggers the Nanog transcription factor. All of these are pluripotency factors, which enable the maintenance of an undifferentiated, self-renewing state [12,49]. What also can be executed by HIFs is reprogramming mature cancer cells toward stemness. In glioma cells, it has been found that the HIF-1α/HIF-2α-Sox2 network manages their dedifferentiation into a CSC-like state, contributing to heterogeneity and further therapy resistance [50].

Apart from activating the above-mentioned Yamanaka pluripotency factors (Oct3/4, Sox2, Klf4, c-Myc), HIFs participate in signal transduction through pathways responsible for undifferentiated cell state maintenance. Through direct interaction with the Notch intracellular domain, HIF-1α is recruited to promoter regions of Notch-responsive genes, subsequent activation of which enhances self-renewal ability and enables preservation of stemness [18]. These properties are also supported through Hedgehog signaling, and hypoxia has been shown to upregulate the expression of key components of this pathway. As a result of hypoxic condition-mediated upregulation of Smoothened (SMO), a G protein-coupled receptor, a ligand-independent activation occurs, leading to GLI transcription factors nuclear translocation and, finally, expression of stemness-associated genes [51,52]. Moreover, HIF-1α is also able to interact with NF-κB and increase growth factor transcription, inducing a further increase in stem-like features through the PI3K/Akt/mTOR pathway. In addition, Akt/mTOR signaling in a positive feedback loop enhances the translation of HIF 1α itself, reinforcing the hypoxic response and supporting stemness [6,12,51].

- EMT as a Process Leading to Phenotypic Heterogeneity

The state of hypoxia is also strictly associated with phenotypic heterogeneity, mostly manifested by cancer cell epithelial-to-mesenchymal transition (EMT), a process in which organized, adherent epithelial-like cells are transformed into motile, loosely connected cells of mesenchymal phenotype. Stabilized HIF-1α, both directly and indirectly, can promote such a phenotypic switch. The association between a hypoxic environment and activation of EMT-related transcription factors (EMT-TFs), such as zinc finger transcriptional repressors Snail, Slug, Twist, and zinc finger E-box binding homebox 1/2 (ZEB1/2), is quite well studied and proven on various cancer types, such as hepatocellular carcinoma (HCC), ovarian cancer, or head and neck squamous cell carcinoma (HNSCC) [53]. The key event in EMT is a cadherin switch, where a loss of E-cadherin—a molecule of a crucial role in the maintenance of cell–cell adhesion and antigrowth signals transduction—in favor of N-cadherin, promoting metastatic behavior, occurs [54]. It has been shown that stabilized HIF-1α, through the direct binding to hypoxia-response elements (HREs) in the proximal Twist gene promoter in the nucleus, leads to its upregulation and direct repression of E-cadherin as a result of histone deacetylation, leading to a closed chromatin conformation [55]. Similarly, Snail binds to E-box elements within the E-cadherin promoter, and subsequently to binding, recruits co-repressor complexes, including histone deacetylases, which, by modifying chromatin structure, silence the gene transcription. The resulting loss of E-cadherin disrupts cell–cell adhesion and epithelial polarity, while concurrent upregulation of mesenchymal genes leads to increased cell motility, invasiveness, and the ability to dissociate from primary tumor sites. Apart from binding to HREs and thereby inducing direct activation of these TFs, HIF-1α also activates histone deacetylases such as HDAC3 that remodel chromatin in favor of Snail expression [56,57,58]. Despite its leading role as an EMT-TF activator, HIF-1α is not the only representative of its family since HIF-2α has been reported to interact with Twist2 in pancreatic cancer, repressing E-cadherin transcription [59]. In addition, HIF-1α upregulates other genes whose products contribute to cancer cell invasiveness, such as IL-8, VEGF, and matrix metalloproteinase-9 (MMP-9), responsible for proteolytic cleavage of vascular basement membranes, clearing the way for mesenchymal-like cancer cells to disseminate [60].

Notably, HIF-1α stabilization influences several key signaling pathways related to EMT, such as the transforming growth factor β (TGF-β) pathway. As observed in ameloblastoma and gastric cancer cells, under hypoxia, TGF-β is upregulated due to HIF-1α-mediated gene expression, intensifying signal transduction through the TGF-β/Smad pathway. The nuclear import of Smad-based complexes, which is a crucial step, enables binding to their regulatory elements in EMT-TFs promoters, driving their expression and shifting the cell’s phenotype toward increased motility and invasiveness [58,61]. TGF-β signaling leads to excessive ROS production, which in connection with Smad-mediated suppression of HIF-ubiquitinating proline hydroxylases, further causes overexpression and nuclear accumulation of HIF-1α, creating a positive regulatory loop [62]. In parallel, Smad-independent TGF-β signaling is activated, including MAPK and PI3K/Akt pathways, leading to cancer cell proliferation and migration [63].

HIF-1α also has a significant effect on the Wnt/β-catenin pathway, which is another major contributor to EMT alongside TGF-β signaling. As a result of Wnt binding to its receptors known as Frizzled, the β-catenin destruction complex is inhibited, and β-catenin itself translocates into the nucleus, whereupon interaction with T-cell factor/lymphoid enhancer factor (TCF/LEF) upregulates the expression of EMT-inducing genes [64]. The binding of HIF-1α to HREs on the tumor suppressor APC gene promoter results in the inhibition of β-catenin phosphorylation and an increase in its intracellular levels, as well as the rate of nuclear entry of β-catenin molecules, promoting EMT. It has also been observed that signal transduction via the Wnt/β-catenin pathway upregulates HIF-1α activity, creating a positive feedback loop [58]. Studies on liver cancer brought the finding that HIF-1α-mediated β-catenin stabilization has a more profound effect on EMT activation than its interaction with TCF4 [65].

Next in the pathways induced by HIF-1α is Notch signaling, engaged in cell proliferation, death, and differentiation. A cross-talk between Notch protein and HIF-1α in EMT activation has been extensively studied in breast cancer cells [66]. It has been found that stabilized HIF-1α not only upregulates the transcription of Notch ligands and receptors by directly binding to HREs in their gene promoters but also physically interacts with the Notch intracellular domain (NICD) to enhance its transcriptional activity. This cooperation leads to the upregulation of Notch target genes such as Hes and Hey family members, which then contribute to the induction of EMT by promoting the expression of EMT-TFs. Notch, in turn, is capable of upregulating Snail expression directly and indirectly by enhancing interaction between HIF-1α and the lysyl oxidase (LOX) gene promoter. Such hypoxia-triggered LOX upregulation leads to Snail protein stabilization, mediating EMT [67]. HIF-1α-mediated Snail stabilization also occurs through the NF-κB signaling pathway, activating IκB kinase β [68]. Furthermore, HIF-1α mediates EMT induction through the Hedgehog pathway, upregulating expression of its components, and via Rho signaling, which regulates cytoskeletal rearrangements [58].

An example of heterogeneity concerning functional state was observed in cells disseminated from the primary tumor and linked by Fluegen et al. with the occurrence of hypoxia. The authors have shown that in breast cancer, MDA-MB-231, and HEp-3 head and neck squamous cell carcinoma lines, post-hypoxic disseminated tumor cells are more prone to enter a dormancy state and, thereby, evade eradication by chemotherapeutics upon establishing metastatic lesions, contributing to the maintenance of residual disease and able to fuel recurrence [69].

- Chemoresistant Cancer Cells

Cancer cells have developed a variety of molecular mechanisms to avoid death induced by chemotherapeutic drugs, which are applied to eradicate the disease. Chemoresistant cancer cells are increasingly recognized as representing a distinct phenotype that significantly contributes to heterogeneity: since these cells can differ markedly from the bulk of the tumor in underlying biology dictating their responses to therapy, they add to the intratumoral diversity [70,71]. The detailed evaluation of how prolonged low oxygen availability influences chemotherapy efficacy is located in Section 4.2.

### 2.3. Heterogeneity in DNA Repair and the Role of Hypoxia

Some variability concerns also the processes implicated in DNA repair. Such heterogeneity may refer to efficacy, pathway preference, and/or regulatory mechanisms applied by cells to detect and correct DNA damage, and is related to differential sensitivity to mutagens and carcinogens. Heterogeneity in DNA repair is known to occur between individuals due to polymorphisms present in the involved genes, but it is also noticeable in tumor cell populations [72].

Since cancer cells during clonal evolution acquire different mutations and are exposed to varying epigenetic modifications, some of the subclones will be endowed with higher DNA repair capacity, while others will exhibit impaired reparation capabilities due to mutations or silencing of key genes controlling such processes. As a consequence, cells with lower repair capacity acquire additional mutations or, as a compensatory mechanism, rely on other pathways, which can be more prone to errors. Both scenarios eventually lead to an increase in the mutational load and traits of genomic instability in particular tumor regions, with the concurrent existence of areas more proficient at DNA repair. Hypoxia, as a crucial contributor to broad transcriptional reprogramming, would also shape the DNA repair capacity of cancer cells. As it will be described in the following chapter of this manuscript, the major consequence of hypoxia on cellular DNA repair mechanisms is the downregulation of high-fidelity DNA repair pathways such as homologous recombination, forcing cells to rely on more error-prone repair mechanisms of NHEJ. As a result, new mutations are acquired, deepening the genomic instability of hypoxic cells [40,73,74].

Oxygen availability varies within tumors; thereby, some regions may retain relatively higher repair function while others suffer severe downregulation. This creates a mosaic of repair proficiency, where cells in more hypoxic niches accumulate mutations that contribute to a large extent to further tumor evolution, promoting heterogeneity and facilitating the development of more aggressive and/ or drug-resistant clones [40,75]. However, more studies are needed to provide accurate and detailed observations regarding the range, extent, and implications of hypoxia-mediated DNA repair heterogeneity.

## 3. Hypoxia-Induced Modulation of DNA Repair Pathways

While hypoxia itself does not cause double-strand breaks (DSBs), it greatly contributes to mutation frequency by modifying the efficiency of different DNA repair pathways [76,77,78,79]. The two main DNA repair mechanisms utilized in DSB are homologous recombination (HR) and non-homologous joining (NHEJ).

The former process involves a homologous sequence found on the sister chromatid corresponding to the lost DNA fragment. Therefore, its occurrence is restricted by its availability and usually concerns the G2 and S phases of the cell cycle. DNA fragments at the breakpoints are initially detected and modified by the MRN complex, consisting of MRE11, RAD50, and NBN, as preparation for undergoing repair. Further, repair proteins such as Rad51, Rad52, and replication protein A (RPA) bind with the overhangs and form a joint molecule between the damaged and undamaged DNA strands. Template-based DNA synthesis occurs, and the completed strands are eventually resolved. Therefore, it is regarded as a high-fidelity repair mechanism. In contrast, in the case of NHEJ, the ends of broken DNA strands are bound together by two Ku70/80 and two DNA-dependent protein kinase catalytic subunit (DNA-PKcs) proteins. The ends are then processed to form a ligation-prone sequence, and the breaks are ultimately repaired by the ligase IV/XRCC4 complex [80].

### 3.1. DNA Damage Response Pathway

Ataxia–telangiectasia-mutated (ATM) and ataxia–telangiectasia and Rad3-related (ATR) kinases are the key transducers of the DNA damage response. They phosphorylate proteins involved in DNA repair via both HR and NHEJ pathways.

ATM is recruited to sites of DNA damage by sensors, among which histone H2AX plays a crucial role. Upon damage detection, H2AX undergoes rapid phosphorylation, which is a key step leading to the recruitment of DSB repair proteins, such as MRN complex, MRE11 nuclease, exonuclease 1 (EXO1), and BLM helicase, to restore DNA integrity at the specific damage site. Further, the damage signal is transduced to the effectors, such as CHK2 and p53. H2AX also activates cell cycle checkpoint kinases that pause cell division to allow for DNA repair [81].

Hypoxia is a condition that activates ATM regardless of the presence of DNA damage due to its ability to induce replication stress, when DNA replication forks are impaired or stalled as a result of compromised activity of ribonucleotide reductase, thereby reducing the deoxyribonucleotide (dNTP) pool. Instead of the MRN complex, which is not involved in hypoxia-mediated ATM activation, ATM interactor (ATMIN) and the histone acetyltransferase Tip60 are essential mediators required for efficient ATM phosphorylation in response to replication stress in hypoxic conditions. Activated ATM phosphorylates CHK2 at Thr68, which, in turn, contributes to HIF-1α stabilization. Downstream effects include cell cycle arrest, chromatin remodeling, enhanced DNA repair capacity, and metabolic reprogramming toward glycolysis, all of which enhance cell survival under low oxygen [82,83,84].

**Figure 3:**
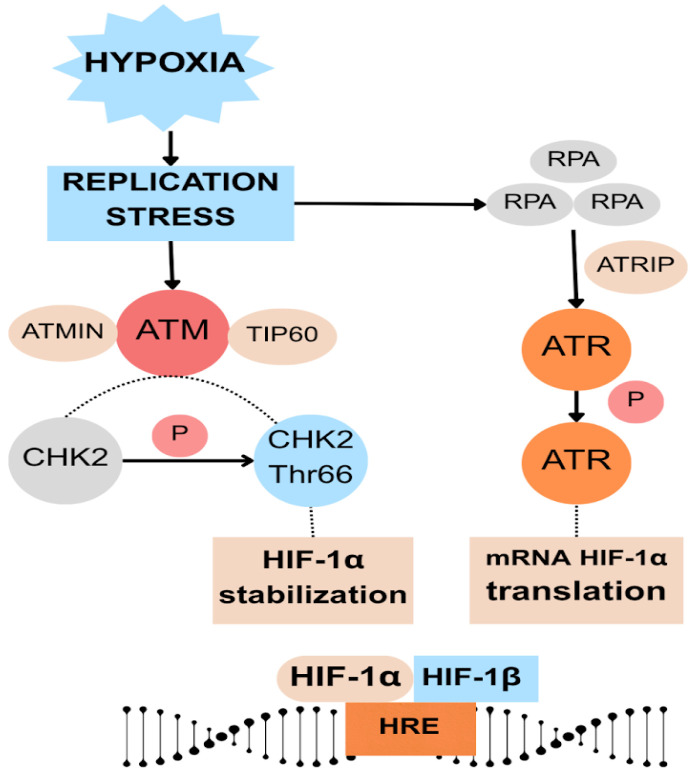
Illustration of the activation of ATM and ATR pathways in response to hypoxia-induced replication stress. ATR is recruited via RPA (Replication Protein A)-coated single-stranded DNA and its co-factor ATRIP. ATM, possibly through a non-canonical mechanism, promotes HIF-1α stabilization via CHK2, while ATR enhances HIF-1α mRNA translation. Together, these responses contribute to the activation of hypoxia-responsive gene expression.

ATR is a central sensor of replication stress, activated upon accumulation of single-stranded DNA (ssDNA). The ssDNA ends, coated by replication protein A (RPA), recruit ATR via its partner ATR-interacting protein (ATRIP), triggering ATR autophosphorylation and full kinase activation [79]. Importantly, in low oxygen availability, ATR enhances translation of HIF-1α mRNA, activating the hypoxic transcriptional program, leading to upregulation of glycolytic metabolism, angiogenesis, and survival pathways under hypoxia (Figure 3) [85]. Loss of ATR through its silencing or inhibition results in increased DSB markers (γH2AX) and higher rates of micronuclei formation after reoxygenation, underscoring its crucial protective role [79]; however, its activity may differ depending on the cell type. The activation involves autophosphorylation and can happen independently of MRN interaction [82]. ATR is recruited primarily by the Ku70/Ku80 complex [79]. Its activation in hypoxia is caused mainly by the influence of hypoxia-induced replication stress. Its downregulation leads to increased cell death under hypoxia/reoxygenation [86].

**Figure 4:**
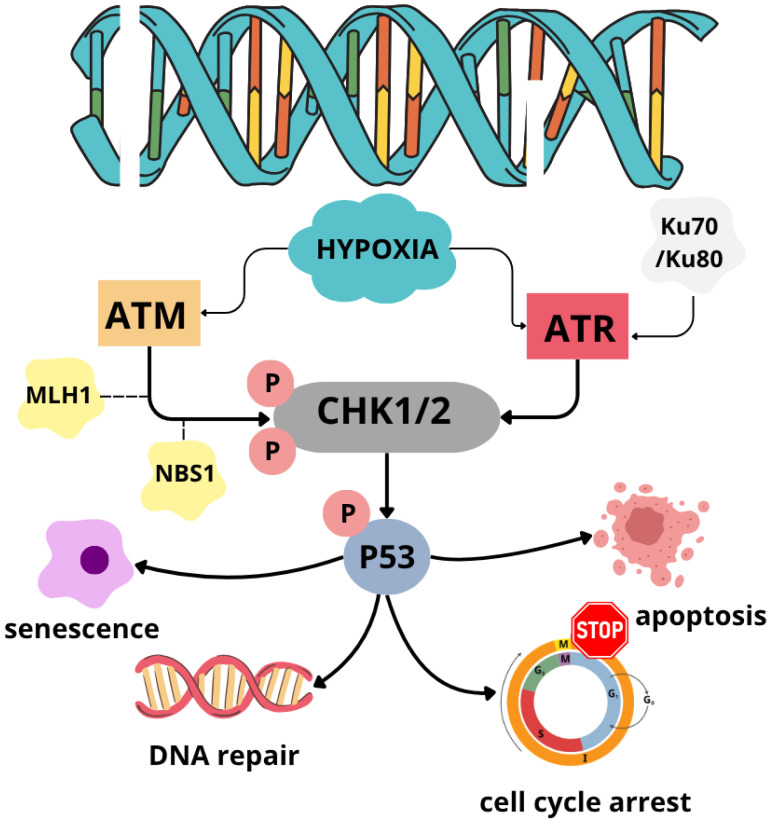
DNA damage response is regulated by key kinases ATM and ATR, which are activated under stress conditions such as hypoxia. These kinases activate checkpoint proteins CHK1/2, leading to the phosphorylation of the tumor suppressor protein p53. In response, P53 initiates cellular processes, including DNA repair, cell cycle arrest, apoptosis, and senescence. Proteins like MLH1, NBN, and Ku70/Ku80 support damage-sensing and repair pathways, maintaining genomic stability.

The activity of ATM/ATR effectors, checkpoint kinase 1 (CHK1), 2 (CHK2), and p53, also increases in hypoxia. CHK2 activation is based on Thr68 phosphorylation in a manner dependent not only on ATM but also MLH1 and NBN genes, encoding the DNA mismatch repair protein Mlh1 and the DSB repair factor nibrin, a component of the MRN complex [87]. Interestingly, despite its role in CHK2 phosphorylation, the Mlh1 protein is downregulated in human cancer cells under hypoxic conditions (Figure 4) [87,88]. Nevertheless, phosphorylation-based CHK2 activation remains dependent on Mlh1 in hypoxic cancer cells [87], which implies its role as a facilitator in ATM-CHK2 interaction rather than directly shaping its enzymatic activity.

### 3.2. Homologous Recombination

The inhibitory influence of hypoxic conditions on the activity level of homologous recombination has been widely documented [89]. The lack of oxygen decreases its functionality through transcriptional, translational, and epigenetic suppression [76]. Lack of proper oxygen supply leads to downregulation of many essential HR genes, including RAD51, RAD51B/C/D, RAD54B, and XRCC2/3, mainly under prolonged hypoxic conditions (>24 h), prevalent in metabolically demanding tumor cells, and regardless of potential cell cycle phase alterations [90]. The downregulation of BRCA1, RAD51, and FANCD2 might occur due to transcriptional repression induced by the E2F4/p130 complex formed as a response to hypoxic stress [77,89].

This downregulation might also result from decreased translational efficiency [77] or translational inhibition of HR and MMR proteins mediated by specific microRNAs [91], whose concentration is raised in hypoxic cells [92]. RAD52, responsible for recruiting RAD51—a key component of HR-based repair, involved in searching for homology and strand pairing—is suppressed by high levels of miR-210 and miR-373. Reversibility of this suppression, achieved by using mi-R antisense inhibition, seems to confirm this dependence [92]. BRCA1 and BRCA2 genes are not only downregulated but also more prone to acquire a variety of mutations in hypoxia [75], which further deteriorates their functionality.

Epigenetic modifications driven by hypoxia include long-term silencing of BRCA1 and RAD51 promoters, with decreased methylation of the fourth lysine residue on DNA packaging protein histone H3 (H3K4) established as a key suppressive factor [93]. The epigenetic regulation of DNA repair might also be mediated by the aggregation of oncometabolites such as fumarate, succinate, or 2-hydroxyglutarate, which are abundant in hypoxic environments [94,95]. These metabolites function as competitive inhibitors of ten–eleven translocations (TET) and histone lysine demethylases (KDM) enzyme families, important epigenetic factors ensuring correct HR pathway trajectory. Inhibiting TET and KDM activity leads to increased DNA and histone methylation, promoting a repressive chromatin state that silences HR-related genes and contributes to HR deficiency [95].

### 3.3. Non-Homologous End Joining

While the high-fidelity HR mechanism seems to be unambiguously downregulated by hypoxia, its influence on the activity of a more error-prone DNA repair pathway, NHEJ, has not been determined as conclusive [76]. At first, only indirect influence was established; suppression of genes favoring the HR-based repair pathway was thought to promote the compensatory increase in NHEJ pathway exploitation in the DSB repair process [89]. Ku70 and Ku80, a heterodimeric protein complex responsible for the recruitment of DNA-PKcs as part of the NHEJ pathway, were determined to either be downregulated or upregulated under varying hypoxic conditions, depending on the severity of oxygen deficiency, type of the affected cell and the exact time of exposure to hypoxic conditions [79].

Mild hypoxia activates DNA-PKcs, important transducers of the NHEJ pathway. Interestingly, elevation in their activity provoked by decreased oxygen supply did not correlate with increased recruitment of DNA repair protein XRCC4 complex with ligase IV, but with increased expression and stabilization of HIF-1α structures [96], the contribution of which to increased NHEJ activity is discussed below. Furthermore, it is implied that while DNA-PKcs might not shape the expression of downstream factors such as Ligase IV, its lack of activation might serve as a physical block for synaptic complex formation, preventing the NHEJ pathway from progressing [97]. Its activation, including increased activation described in hypoxia, leads to autophosphorylation, which then physically allows for the LIGIV-XRCC4 complex to bind to the damaged DNA strands.

Nevertheless, the literature presents contradictory evidence. The downregulation of DNA IV ligase, as well as downregulation and mutation of XCC4 [78], both essential components for NHEJ pathway activity, have been reported under hypoxic conditions in various cancer cells. Additionally, hypoxia inhibits the expression of the PTEN tumor suppressor, which decreases the activity level of the NHEJ repair system [98]. Some cellular features present in hypoxic conditions are shown to influence both HR and NHEJ in the same manner, primarily by modifying the common elements for both pathways. Inhibition of TET and KDM enzymes by oncometabolite accumulation prevents both HR and NHEJ components from working properly [95].

### 3.4. HIF-1α

**Figure 5:**
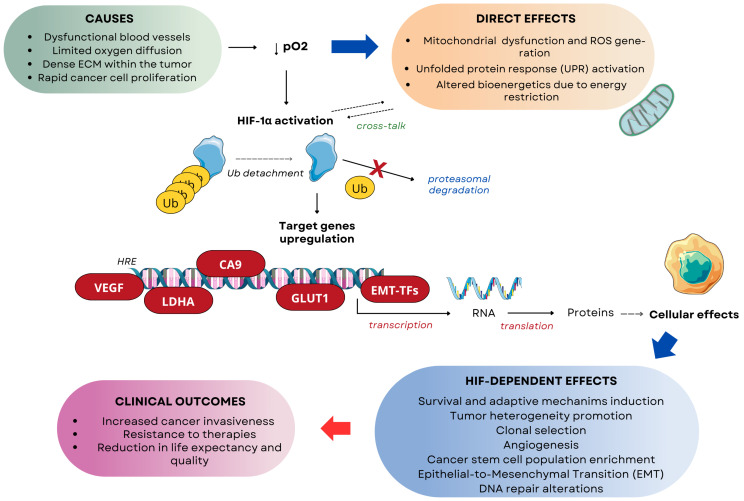
The simplified representation of direct and HIF-dependent effects driven by hypoxic conditions in cancer cell microenvironment. Insufficient supply of oxygen induces mitochondrial dysfunction, UPR, and reprograms cellular bioenergetics toward glycolysis. HIF-1α, no longer degraded as a result of ubiquitin detachment in hypoxia, upregulates various target genes interacting with their hypoxia-response elements (HREs), supporting cancer cells in proliferation, survival, and increasing their invasiveness. It leads to resistance to a range of anticancer therapies, which is associated with treatment failures, recurrences, and overall reduces patient’s life expectancy as well as quality.

The proper evaluation of hypoxia-induced factors (HIF) role is essential for comprehending the complexity of cellular response in hypoxia. HIF-1α, a key representative of hypoxia-induced factors residing in the cytoplasm, is quickly subjected to proteasomal degradation initiated by hydroxylation performed in oxygen-dependent degradation domains, mediated by prolyl hydroxylases (PHDs) in a condition of sufficient O_2_ cellular supply. Once proline residues are hydroxylated, the von Hippel–Lindau (pVHL) tumor suppressor protein, a part of an E3 ubiquitin ligase complex, binds to HIF-1α, leading to its polyubiquitination. Under hypoxic conditions, the early degradation is prevented. Since PHDs require molecular oxygen to maintain their activity, hydroxylation of HIF-1α proline residues under low O_2_ availability is highly reduced. As a result, pVHL does not recognize HIF-1α, allowing it to escape being labeled for proteasomal degradation and, thereby, accumulate in the cytoplasm. Stabilized HIF-1α is transported to the nucleus, where it connects with HIF-1β. The newly formed HIF dimeric structure, after interacting with its coactivators, initiates transcription of HIF target genes by binding to specific DNA sequences known as hypoxia-response elements (HRE) [79,99]. The collective effect of hypoxic conditions on cancer cells is presented in Figure 5.

HIF proteins, master regulators of molecular response to hypoxia, shape the activity of the majority of DNA repair systems [100]. HIF-1α competes with transcription activator c-Myc for Sp1 binding sequence in the gene promoter, which causes significant downregulation of MSH2 and MSH6 genes, encoding mismatch repair proteins. In the form of a heterodimer, their products function as important tumor suppressor factors involved in many DNA repair forms, including homologous recombination and base excision repair. Under prolonged hypoxia, when c-Myc subsides to HIF-1α, which binds to the promoter sequence, transcription of both MSH2 and 6 is repressed, increasing mutagenesis and leading to the situation where replication errors are no longer efficiently detected or repaired. In addition, MMR activity is also impaired under sustained low-oxygen conditions, reducing the efficiency of HR and further increasing mutation rates due to compensatory shifts toward more error-prone repair systems [40,101]. Moreover, HIF-1α overexpression increases β-catenin nuclear translocation, leading to β-catenin-regulated NHEJ transcriptional activation [102].

The expression of certain microRNAs, which were previously mentioned as inhibitors of the translation of HR and MMR proteins, is also raised in a HIF-1α-dependent way [92]. A better understanding of HIF influence allowed for clearer insight into the functioning of the NHEJ repair pathway. Research proves that downregulation of HIF-1α, achieved by small interfering RNA, caused a significant decrease in XRCC4, which serves as a cofactor of the catalytic subunit DNA ligase IV, crucial for the NHEJ system to work [102]. Moreover, HIF-1α overexpression increases β-catenin nuclear translocation, leading to β-catenin-regulated NHEJ transcriptional activation [102].

The von Hippel–Lindau tumor suppressor (pVHL) is a major factor regulating HIF-1α activity. VHL gene’s product serves as a ligase that ubiquitinates HIF-1α, leading to its degradation by the proteasome when oxygen is available. In VHL-deficient renal cancer cells, the expression of genes involved in double-strand breaks repair by homologous recombination and mismatch repair was found to be downregulated, while the NHEJ genes’ expression was determined to be unaffected by the VHL loss, achieving approximately the level as in the cells with the correct functioning of the VHL gene [77]. These findings seem to further confirm the existence of HIF-1α-mediated imbalance between HR and NHEJ repair systems, favoring the latter. Nevertheless, it is important to emphasize that VHL inactivation might have consequences other than HIF-1α accumulation [103], which might also be relevant in changing the usage frequency of each DNA repair pathway. Furthermore, VHL inactivation might also occur in normoxic conditions, resulting in the activation of similar metabolic pathways [104].

It is important to state that despite its status as the master regulator of hypoxic adaptation, some hypoxia-induced processes remain unaffected by the influence of HIF. Hypoxic downregulation of BRCA1 and RAD51, mediated by transcription repressors E2F4/P130, was found to happen regardless of HIF-1α activity [90].

### 3.5. Biomarkers of Hypoxia

Hypoxia is a hallmark of the tumor microenvironment and has profound implications for cancer progression, therapeutic resistance, and immune evasion. To evaluate hypoxic status, a range of molecular and chemical biomarkers has been identified. Among endogenous markers, HIF-1α serves as a central transcriptional regulator activated under low oxygen tension. Its downstream targets, including carbonic anhydrase IX (CAIX), glucose transporter 1 (GLUT1), vascular endothelial growth factor (VEGF), and lysyl oxidase (LOX), are frequently used to infer hypoxic signaling through immunohistochemical or gene expression analyses [105].

Non-coding RNAs, particularly miR-210, have emerged as promising hypoxia-regulated biomarkers detectable in tissue and circulating samples, offering potential for non-invasive assessment [106,107]. Exogenous bioreductive compounds such as pimonidazole and EF5 are used experimentally and clinically to directly label hypoxic cells, enabling spatial mapping of hypoxia via imaging or histological methods [108]. The integration of multiple hypoxia biomarkers can enhance diagnostic accuracy and may support the stratification of patients for hypoxia-targeted therapies. These major biomarkers, their biological functions, and clinical relevance are presented below (Table 1).

## 4. Impact of Hypoxia on Cancer Therapy Efficacy

### 4.1. Radiotherapy

Hypoxia influences the effectiveness of radiotherapy, affecting the treatment outcome [133]. Normoxic tumor cells display a threefold increase in radiation sensitivity compared to anoxic cells [134]. Studies show that to inflict equivalent damage to that in normoxia, there is a need for two–three-fold higher doses of irradiation in hypoxia [135]. The difference in radiation sensitivity is measured by the OER (Oxygen Enhancement Ratio), which is higher in hypoxic environments [136]. All of the six “R’s” commonly used to describe the mechanisms through which radiotherapy affects cancer cells have been proven to be disturbed by hypoxia. The “six Rs” comprise Repair, Redistribution, Repopulation, Reoxygenation, Radiosensitivity, and Reactivation [137].

Ionizing radiation causes DNA damage in exposed tumor tissue, which leads to cell death. Radiation creates unstable free radicals in biological matter. These radicals damage cells by reacting with oxygen. In hypoxia, lack of molecular oxygen hinders the apoptotic effect of the free radicals [135]. Moreover, hypoxic tumor cells can chemically repair radiation-induced DNA damage through the removal of hydrogen from free sulfhydryl groups after the radiation-induced DNA damage. In normoxic cells, radiation creates stable peroxides that hinder DNA repair [136].

Various mechanisms enable tumor cells to adapt to hypoxia and attenuate radiotherapy-induced damage. Stabilization and activation of HIF transcriptional factors under hypoxia lead to an increase in oxygen transport, angiogenesis, and proliferation. Antioxidant capabilities are enhanced through glycolytic metabolism. This leads to vascular protection and recovery of blood and nutrient supply, reducing the effectiveness of radiotherapy treatment as well as facilitating post-irradiation recurrence of tumors through the promotion of angiogenesis and vasculogenesis [138]. HIF-1α also modulates the tumor cell cycle, preventing cells from entering G2/M phase, which is characterized by increased radiosensitivity [136].

Other, HIF-independent mechanisms also facilitate cancer cell adaptation to hypoxia [139]. Cellular adaptation under hypoxia, such as increased ROS production (and indirect increase in ROS scavengers), genomic instability, clonal selection, replication stress, and diminished direct physicochemical effects of irradiation in hypoxic tumors, increases tumor cell resistance to radiotherapy. Hypoxia prevents apoptosis despite radiation-induced damage through the induction of expression of anti-apoptotic genes (such as IAP-2) as well as downregulation of pro-apoptotic proteins [133,136]. As a significant cellular stressor, severe hypoxia triggers the unfolded protein response (UPR) and inhibits global mRNA translation, reducing cellular activity and making cells less susceptible to damage from radiation [140].

### 4.2. Chemotherapy Inefficacy and Chemoresistance

Hypoxic conditions are placed among the bases of chemoresistance: they were found contributing to both the onset of this phenomenon and to support its maintenance, in pleiotropic manners. The major mechanism is based on the ability of HIF-1α and HIF-2α to upregulate the expression of genes encoding efflux pumps, such as P-glycoprotein (P-gp), multidrug resistance-related protein 1 (MRP1), or breast cancer resistance protein (BCRP), due to binding with their HREs and controlling transcription [60]. P-gp is considered the main villain of cancer multidrug resistance. Along with MRP1 and BCRP, it belongs to the family of ATP-binding cassette (ABC) membrane transporters, which actively remove a broad range of xenobiotics, including chemotherapeutics, from the cytoplasm to the extracellular environment. In consequence, drug concentrations inside the cancer cell are too low to exert a therapeutic effect [141]. For example, HIF-1α-mediated upregulation of P-gp and MDR1 was found to be associated with the resistance of gastric cancer cells to doxorubicin and vincristine [142].

Another HIF-mediated mechanism of supporting cell survival upon exposure to chemotherapeutics is related to DNA repair. While chronic hypoxia generally impairs high-fidelity DNA repair pathways such as homologous recombination and mismatch repair, contributing to genomic instability, HIF-1α activation may transiently enhance alternative or compensatory DNA repair mechanisms to support cell survival under stress, thereby contributing to chemotherapy-induced death evasion [143]. This context-dependent regulation highlights the dual and sometimes opposing roles of hypoxia in genome maintenance. Indeed, a number of cell-based and in vivo studies revealed that HIF-1α increases the expression and activity of poly (ADP-ribose) polymerase-1 (PARP-1), a DNA repair protein complementing XP-A cells (XPA) and XPD helicase. It occurs both through direct transcriptional activation of these enzymes and by altering chromatin structure through the induction of chromatin remodelers, which facilitates rapid recruitment of PARP 1 to DNA lesions and repair [40,143]. Additionally, activation of antiapoptotic proteins and pathways occurs upon induction by HIF-1α, contributing to cancer cell survival and weakening the response to therapeutics—higher doses of the drug are tolerated by cells. This has been proven by HIF-1α knockout studies, where cells deprived of this factor presented downregulation of surviving with upregulation of proapoptotic Bax and caspases 3 and 8 [144]. An important finding was reported by Rohwer et al., who showed that in gastric cancer cells, HIF-1α suppresses p53 activation upon exposure to 5-fluorouracil. This suggests another mechanism of HIF-1α-mediated impairment of chemotherapy-induced apoptosis, dependent on a functional p53 pathway [145]. Moreover, hypoxia is an activator of autophagy—a process in which damaged or unnecessary cellular components are degraded and recycled. In the context of chemotherapy, autophagy can be upregulated as a survival response, allowing cancer cells to endure the stress induced by drugs. This recycling mechanism provides energy and substrates needed for cell survival, effectively enabling the cancer cells to resist the cytotoxic effects of the chemotherapeutic agents [146]. HIF-1α serves as a key regulator of hypoxia-induced autophagy, promoting survival of cancer cells in the presence of cytotoxic compounds and leading to the development of resistant clones [143].

There is also a link between HIF-1α, metabolic alterations, and chemoresistance. As a potent transcription factor, HIF-1α intensifies glycolysis, upregulating its key enzymes and transporters such as GLUT1, HK2, fructose bisphosphatase (FBP), pyruvate kinase M2 (PKM2), and LDHA. This metabolic switch, as aforementioned, leads to ROS production and apoptosis evasion, reducing the effects of chemotherapeutics and thereby supporting resistance development [147]. In addition, a study on lung cancer cells A549 revealed that hypoxic conditions activate another transmembrane enzyme, carbonic anhydrase IX (CAIX), which is able to neutralize intracellular acidosis. In turn, extracellular acidosis is induced due to export of H^+^ and HCO_3_^−^ outside the cell. This effect contributes to resistance to vinorelbine, which—as a weak base—becomes protonated and inactivated [148]. Development of resistant clones is also related to alterations in mitochondrial metabolism and functionality. HIF-1α, as a mitophagy inducer, supports the recovery of ATP and reducing factors, allowing for an increase in ABC transporter activity and repairing chemotherapy-induced damage to macromolecules. This mechanism was associated with resistance to 5-fluorouracil, gemcitabine, and cisplatin. It was also already mentioned that OXPHOS impairment due to the Warburg effect in cancer cells leads to ROS accumulation and maintenance of its sub-cytotoxic levels. The adaptive response, enabling cell survival, is guaranteed by the Nrf2 (nuclear factor erythroid 2-related factor 2)-dependent pathway, which, apart from activating antioxidant enzymes, upregulates MRP1. Both ways contribute to chemoresistance: the chemotherapy-induced ROS neutralization attenuates oxidative damage induced by chemotherapeutics, while MRP1 significantly reduces the retention of drugs inside cancer cells [60]. Also, chemoresistant ovarian and prostate cancer cells regained sensitivity to cisplatin upon HIF-1α knockout, indicating that it has a great contribution to the development of resistant phenotypes [147].

Moreover, many chemotherapeutic agents require molecular oxygen for maximal activity, which often includes the generation of ROS, aiding in inducing lethal DNA damage in cancer cells. In hypoxic regions of solid tumors, these drugs become less effective because of the low O_2_ levels impeding their activation. The most well-known example of such chemotherapeutics would be the group of anthracyclines, including doxorubicin (DOX) and daunorubicin. Their mechanism of action is based on DNA intercalation and, due to the presence of quinone residues, ROS production. In the presence of NADPH and O_2_, they form semiquinone radicals that reduce O_2_ to superoxide (O_2_•^−^), leading to the formation of hydrogen peroxide (H_2_O_2_) and hydroxyl radicals (•OH), which trigger DNA strand breaks, lipid peroxidation, and cell membrane disruption. Hypoxia impairs this redox cycle, leading to less ROS production and, therefore, increased cancer cell survival upon treatment [149,150,151]. Another oxygen-dependent chemotherapeutic is bleomycin, which binds DNA and, in the presence of Fe^2+^ and O_2_, generates DNA strand breaks via the formation of hydroxyl radicals. Specifically, Fe^2+^–bleomycin chelates O_2_ to form a Fe-OOH intermediate, which tears hydrogen atoms off deoxyribose, causing both single- and double-strand breaks. Without sufficient O_2_, the Fe-bleomycin complex cannot cycle through its high-valent oxygenated intermediates, drastically reducing DNA cleavage [152].

What is also worth mentioning in the context of chemoresistance development is that in hypoxic conditions, chronic endoplasmic reticulum (ER) stress leads to activation of the unfolded protein response (UPR) in tumor cells—a mechanism protecting cells from protein homeostasis disruption. In the hypoxic environment, ATP production is impaired, as is the proper folding of polypeptides synthesized in the ER, which is an energy-consuming process. The following accumulation of misfolded proteins triggers the UPR, which induces a shift toward an adaptive phenotype by suppressing apoptosis, enhancing cytoprotective autophagy, and increasing the capacity of cells to neutralize ROS. Moreover, the downstream activation of IRE1α/XBP1 signaling promotes the upregulation of genes for ER-associated degradation, transcription of the MDR1 gene, and stem-like features in cancer cells, altogether contributing to the development or deepening of resistance to chemotherapeutic agents [153,154,155,156].

### 4.3. Immunotherapy

Tumor hypoxia plays a multifaceted role in shaping the immune landscape of the tumor microenvironment. It is well established that hypoxia can promote immunosuppression by upregulating inhibitory molecules such as PD-L1, expanding regulatory T cells, and recruiting myeloid-derived suppressor cells. However, emerging evidence suggests that hypoxia may, in certain contexts, enhance immune cell infiltration, including that of cytotoxic T cells. One proposed mechanism is that hypoxia-induced genomic instability increases tumor mutation burden (TMB), leading to the generation of neoantigens that can stimulate T cell recruitment. This dual role highlights the complex interplay between oxygen availability, tumor immunogenicity, and antitumor immunity [157,158].

Hypoxia reduces the effectiveness of immunotherapy and attenuates the immune system’s response against cancer cells. Accumulation of HIF-1α has a significant impact on immune checkpoint ligand expression, such as programmed death-ligand 1 (PD-L1), through its direct binding to the HRE sequence in the PD-L1 promoter. PD-L1, located on the tumor cell surface, binds to its receptor PD-1 on T cells, inhibiting T-cell receptor (TCR)-mediated activation of IL-2 production and T-cell proliferation. Therefore, the cytotoxic effect is hampered, and T-cell exhaustion occurs [159,160]. Such abundance of PD-L1 in hypoxic regions contributes to lower efficacy of PD-1/PD-L1 inhibitors and reduces overall response rates [157].

As mentioned in the previous sections, hypoxia promotes glycolysis, resulting in acidification due to high levels of lactate being secreted into the extracellular milieu. Low pH also contributes to increased PD-L1 expression and creates a favorable environment for interaction between the ligand and its receptor, PD-1. This effect is associated with the inefficacy of immune checkpoint inhibitor (ICI) therapy targeting PD-1. It has been shown that acidic conditions can suppress CD8^+^ T-cell cytotoxicity by >60% in comparison to pH 7.4, with reduced TCR–driven calcium flux and IFN-γ release. Moreover, a lack of glucose along with tryptophan and arginine activates autophagy of T cells [161,162].

Another consequence of the cancer cell metabolic switch toward glycolysis is adenosine overproduction. Extracellular adenosine is known to induce immunosuppression, impairing T-cell proliferation and cytokine secretion, while promoting Treg cell differentiation. This effect is driven by direct HIF-1α-induced upregulation of CD39 and CD75 ectonucleotidases, both on cancer cells and T lymphocytes. Their activity leads to the accumulation of adenosine, which further binds A_2_A receptors on T cells and initiates cAMP–PKA signaling, upregulating inhibitory cytokines like TGF-β and checkpoint receptors [163]. Hypoxia also inhibits adaptive immune system cells through impaired dendritic cell maturation and antigen presentation [1,164].

Immunotherapy based on chimeric antigen receptor T-cells, known as CAR-T, is considered one of the most promising cancer treatments developed and introduced to clinics in recent years. However, tumor hypoxia imposes several barriers that reduce CAR-T cell efficacy, including metabolic stress, exhaustion, and impaired tumor infiltration. Restricted oxygen concentration, elevated lactate levels, and low glucose together impair proliferation and drive early exhaustion of lymphocytes. For example, a 2020 study has shown diminished expansion of both transduced (CD-19-targeting) and non-transduced T cells by nearly 50%, and shifted their differentiation from central memory toward an effector memory phenotype, which is associated with reduced lifespan [165]. In addition, CAR-T—like non-engineered T cells—are prone to the induction of PD-1 or another immune checkpoint receptor, LAG-3, expression in hypoxic conditions, leading to a reduction in their functionality [166]. There is also evidence that neoangiogenesis, driven by insufficient oxygen supply to cancer cells under hypoxia, reduces CAR-T cytolytic function. Not only do abnormal, disorganized blood vessel arrays limit CAR-T cell infiltration into the tumor tissue, trapping them in the perivascular niches, but also signaling molecules that participate in angiogenesis—with the main contribution of vascular endothelial growth factor (VEGF)—exhibit direct immunosuppressive effects. In solid tumor models, VEGF inhibition improved CAR-T efficacy two-way: through reversing immunosuppression and improving vessel architecture, permitting better CAR-T expansion and functionality [167,168,169].

## 5. Therapeutic Strategies Targeting Hypoxia in Cancer

Several therapeutic strategies aim to overcome hypoxia-mediated resistance and improve cancer treatment outcomes, including hypoxia-activated prodrugs (HAPs), oxygenation therapy, and HIF-1 inhibitors.

### 5.1. Hypoxia-Activated Prodrugs (HAPs)

Hypoxia-activated prodrugs (HAPs) are bioreductive drugs that are selectively activated under hypoxic conditions, enabling them to specifically target hypoxic tumor regions. These prodrugs are typically inactive in well-oxygenated tissues, which is their important feature in minimizing systemic toxicity [141]. Despite the conceptual elegance of HAPs, their clinical success has been limited, and further research is needed to optimize their use. Challenges include limited diffusion into hypoxic tumor regions and potential for off-target effects [170,171,172,173,174]. Compounds with the greatest therapeutic potential are characterized below in Table 2.

#### 5.1.1. Tirapazamine

Tirapazamine is a benzotriazine dioxide that undergoes bioreduction by cytochrome P-450 in hypoxic cells, generating cytotoxic radicals that cause DNA damage, interrupt cell cycle progression, and induce apoptosis. The effectiveness of tirapazamine in counteracting hypoxia was proved many years ago [190], showing promising results in preclinical studies; however, there were difficulties in achieving the same results in clinical trials. Nevertheless, the latest data are promising for TPZ in cancer therapy [175,176]. Transarterial embolization (TAE) is a method of treatment in non-operative patients with hepatocellular cancer (HCC)—it induces ischemia and necrosis; however, the effect is not seamless in all areas, thus leading to the survival of cancer cells and the development of hypoxia regions. As was described above, hypoxia is a major driving factor for tumor progression; thereby, it leads to relapse. The first research group found that administration of tirapazamine alongside TAE in non-operative patients resulted in aborted progression in 80% of patients after six months and 84% overall response rate whereas in the latter research, no further exacerbation in 72.6% of the study group after 6 months, and distinct regression in 64% of patients was observed. Importantly, no serious adverse effects were noticed in either study. Furthermore, the size of lesions did not have any major impact on the efficacy of this method of therapy, compared to trans-arterial chemoembolization (TACE) only, which shows diminished efficiency in patients with lesions over 4 cm [175,176,177].

#### 5.1.2. Evofosfamide

Evofosfamide (TH-302) is a hypoxia-activated prodrug based on a 2-nitroimidazole trigger that has shown strong hypoxia-specific toxicity in vitro across various human cancer cell lines, and significant anti-tumor activity in vivo in several xenograft models [191]. However, it narrowly failed to meet the primary endpoint of improved overall survival, potentially due to inadequate patient selection strategies [192]. The lack of T-cell infiltration in hypoxic tumor regions has been suggested as a contributor to immunotherapy resistance, leading to the idea that targeting hypoxia with evofosfamide (EVO) might improve responses to immune checkpoint inhibitors (ICIs). In a syngeneic TRAMP-C2 prostate cancer model in mice, treatment with EVO resulted in increased blood vessel density, a decrease in hypoxic areas, and greater infiltration of CD3+ T-cells into the tumor. When combined with anti-PD-1 and anti-CTLA-4 therapy, the expansion of myeloid-derived suppressor cells (MDSCs)—typically abundant in hypoxic zones—was significantly reduced. This combination led to enhanced CD8+ T-cell proliferation and cytotoxicity, resulting in tumor clearance in the majority of treated mice with an 82% survival rate. In a related TRAMP transgenic mouse model of spontaneously arising prostate tumors, EVO similarly enhanced ICI efficacy by boosting CD8+ T-cell responses and reducing MDSC proliferation [193]. This synergistic effect between EVO and ICIs has also been observed in other tumor types, such as head and neck squamous cell carcinoma (HNSCC), where the combination of EVO and anti-CTLA-4 therapy significantly improved survival compared to immunotherapy alone [194]. These findings provide proof-of-concept for combining HAPs with ICIs, which further leads to a Phase I clinical trial assessing the safety of EVO with ipilimumab in advanced solid tumors, including pancreatic, melanoma, prostate, and HNSCC (NCT03098160). Among 21 immunotherapy-refractory patients, the combination achieved a 17% overall response rate and an 83% disease control rate across different dosing levels. Responders exhibited peripheral T-cell expansion, greater T-cell and dendritic cell infiltration into hypoxic tumor regions, and a reduction in immunosuppressive tumor-associated macrophage (TAM) activity. Encouraged by these results, ImmunoGenesis announced in late 2020 the initiation of a Phase 2 trial exploring evofosfamide in combination with both anti-CTLA-4 and anti-PD-1 therapies for patients with castration-resistant prostate cancer, pancreatic ductal adenocarcinoma, and HPV-negative HNSCC [195]. In recurrent glioblastoma, the objective “best response” (complete response plus partial response as per RANO criteria) was observed in 4 (17.4%) of 23 patients treated overall with EVO. Disease control (complete response, partial response, and stable disease) was observed in 14 (60.9%) of the 23 patients [182]. During the second clinical trial, 33 patients with bevacizumab-refractory GBM received EVO 670 mg/m2 in combination with bevacizumab 10 mg/kg IV every 2 weeks. A total of 12 patients (36%) suffered grade three–four drug-associated adverse events (AE); no grade 5 AE were reported. Of the 33 evaluable patients, best response was PR in 3 (9%), SD in 14 (43%), and PD in 16 (48%), with responses confirmed by a second reviewer. The median time to progression of disease was 53 days (95% CI 42–113), and the median time to death was 129 days (95% CI 86–199 days). Progression-free survival at 4 months (PFS-4) on Evo-Bev was 31%, which was a statistically significant improvement over the historical rate of 3%. The median overall survival of patients receiving Evo-Bevacizumab was 4.6 months (95% CI 2.9–6.6) [183].

In preclinical non-small cell lung cancer models a statistically significant difference from the vehicle treatment animal group was only found in the EVO-treated groups, including EVO 50 mg/kg monotherapy, EVO 25 and 50 mg/kg combination therapy groups, with the increase in lifespan of 100%, 84%, and 116%, respectively. 50 mg/kg EVO in combination with docetaxel significantly prolonged the survival time compared with ifosfamide 120 mg/kg in combination with docetaxel (*p* < 0.001). Also, EVO 50 mg/kg did not significantly reduce the same blood cell counts compared with the vehicle-treated animals. Compared to ifosfamide at 30 mg/kg, which did not exhibit any antitumor activity, EVO at 50 mg/kg yielded 56% Tumor Growth Inhibition (TGI), which was consistent with the previous data. Interestingly, the combination treatment of EVO and ifosfamide enhanced antitumor activity in this model, with a TGI of 75% [184]. In another study, fifty-nine patients initiated therapy, 31 received the combination of EVO and dexamethasone, and 28 received the combination of EVO, bortezomib, and dexamethasone. The maximum tolerated dose was established at 340 mg/m^2^ EVO + dexamethasone with dose-limiting mucositis at higher doses. For the combination of EVO, bortezomib, and dexamethasone, no patient had dose-limiting toxicity (DLT), and the recommended phase II dose was established at 340 mg/m^2^. Responses included one very good partial response (VGPR), three partial responses (PR), two minor responses (MR), 20 cases of stable disease (SD), and 4 cases of progressive disease (PD) for EVO + dexamethasone and 1 complete response (CR), 2 PR, 1 MR, 18 SD, and 5 PD EVO + bortezomib + dexamethasone. Disease stabilization was observed in over 80% and this was reflective of the prolonged overall survival of 11.2 months [185].

In pancreatic cancer research, the profile of combining EVO with gemcitabine (GEM) and nab-paclitaxel (nP) in human pancreatic ductal adenocarcinoma (PDAC) xenograft models in mice was investigated. The addition of EVO to GEM and nP significantly enhanced the antitumor activity in PDAC xenograft models. In the Hs766t, MIA PaCa-2, PANC-1, and BxPC-3 models, EVO as a monotherapy exhibited modest antitumor effects, with TGI ranging from 27% to 82%, while the G + nP doublet showed TGIs from 85% to 107%. The triplet of G + nP + EVO led to a further increase in TGI, with the MIA PaCa-2 and PANC-1 models showing TGIs of 120% and 110%, respectively. Kaplan–Meier survival analysis revealed a median time (MT) to reach a tumor size of 1000 mm^3^ of 65 days for the triplet in the Hs766t model, significantly longer than G + nP (50 days, *p* < 0.05). The complete response (CR) rate in the PANC-1 model was 100% for the triplet, compared to 60% for the doublet (*p* < 0.05). Histological analysis indicated that the triplet induced significant tumor necrosis (64%) and apoptosis, as evidenced by increased Terminal uridine deoxynucleotidyl transferase-mediated dUTP nick end labeling-positive cells (8.0 ± 0.3 vs. 0.8 ± 0.1 in vehicle, *p* < 0.05). There was no additional toxicity observed in the triplet group, as body weight loss and hematological parameters were similar to the G + nP group [186].

The last disease to date, toward which the efficacy of EVO was assessed in preclinical and clinical studies, is advanced leukemia. In this Phase I study, the maximum tolerated dose (MTD) for EVO was determined to be 460 mg/m^2^ for the daily intravenous infusion (Arm A) and 330 mg/m^2^ for the continuous infusion (Arm B). The dose-limiting toxicities (DLTs) primarily included grade 3 mucositis. Hematologic adverse effects were mainly neutropenic fever (45% in Arm A, 18% in Arm B), with some instances of thrombocytopenia (5%) and anemia (8%) in Arm A. In terms of efficacy, this study showed that 6% of patients achieved an objective response, including two complete responses (CR), one complete remission with incomplete count recovery (CRi), and one partial response (PR). Additionally, 13 patients had stable disease (SD), and two patients had complete resolution of leukemia cutis. Hypoxia biomarkers revealed a significant presence of pimonidazole-positive cells in bone marrow, with seven of eight patients showing >40% pimonidazole-positive cells. Despite transient cytoreduction in circulating blasts (median 67% decrease), most patients experienced disease progression by Cycle 2 (NCT01149915) [187].

#### 5.1.3. CP-506

CP-506 is a second-generation derivative of PR-104—a phosphate pre-prodrug of the dinitrobenzamide nitrogen mustard HAP PR-104A, specifically designed to avoid activation under oxygen-rich (aerobic) conditions by the enzyme aldo-keto reductase 1C3 [187]. Structurally, CP-506 is a nitrogen mustard-based prodrug that remains inactive when oxygen levels are normal. This inactivity under normoxic conditions is due to a nitro group attached to an aromatic ring, which strongly pulls electron density away from the nitrogen mustard component. As a result, the nitrogen mustard cannot effectively form DNA crosslinks because its non-bonding electrons are less available [196]. CP-506 demonstrated its hypoxia-selective activation and cytotoxicity across various in vitro and in vivo models. CP-506 metabolism was inhibited by trace oxygen, with maximal consumption occurring under anoxia, whereas cytotoxicity was progressively inhibited by increasing O_2_ concentrations. CP-506 significantly reduced tumor hypoxia in xenograft models, with increased tumor control at doses up to 800 mg/kg. The study group observed significant improvement in tumor regression compared to the control group [173]. The effects of CP-506 were assessed in clinical trials in combination with radiation on tumor control in two human head and neck squamous cell carcinoma (HNSCC) xenografts: FaDu and UT-SCC-5 models. In the FaDu model, CP-506 significantly increased local tumor control after single-dose irradiation (62% vs. 27%, *p* = 0.024), with an enhancement ratio of 2.3. However, in UT-SCC-5, CP-506 showed only marginal efficacy (*p* = 0.065), indicating less sensitivity. The hypoxic volume (HV) was significantly reduced in FaDu tumors after CP-506 treatment (*p* = 0.038), while it was marginally increased in UT-SCC-5 (*p* = 0.07). DNA damage, measured by staining phospho-histone H2A.X (Ser139) (γH2AX) expression, was significantly higher in FaDu tumors (*p* = 0.009) but not in UT-SCC-5, confirming FaDu’s higher sensitivity to CP-506. The expression of oxidoreductases was significantly increased after CP-506 treatment (*p* < 0.05), indicating potential involvement in its activation. These findings highlight the variable therapeutic responses depending on tumor type and the potential for CP-506 to improve treatment outcomes in hypoxic tumor areas, with particular promise for hypofractionation schedules [181].

#### 5.1.4. Tarloxotinib

Tarloxotinib is a hypoxia-activated prodrug designed to harness tumor hypoxia. Because tarloxotinib acts by inhibiting the epidermal growth factor receptor (EGFR or HER) family proteins, its effectiveness depends on the presence of HER1–4 in tumor cells. Tarloxotinib-E, the active metabolite of the hypoxia-activated prodrug tarloxotinib, showed significantly greater potency than established EGFR inhibitors, such as afatinib, gefitinib, and osimertinib. Tarloxotinib-E inhibited EGFR, HER2, HER3, and HER4 with a 1 μM concentration, while the Cmax for afatinib in patients was only 38 ng/mL (78 nM). In patient-derived cell lines with EGFR exon 20 insertions (CUTO14, CUTO17, CUTO18), tarloxotinib-E was 10-fold more potent in HER2 models compared to EGFR exon 20 models. Xenograft models treated with tarloxotinib-E demonstrated significant tumor regression, with sustained intratumoral concentrations of the drug above 1 μM for up to one week. In contrast, afatinib showed no TGI at the human equivalent dose. Additionally, a patient with metastatic non-small cell lung cancer (NSCLC) harboring a HER2 exon 20 insertion mutation showed an objective tumor response with minimal on-target EGFR-related toxicity. Overall, tarloxotinib-E demonstrated a marked anti-tumor effect with lower systemic toxicity compared to other EGFR and HER2-targeted therapies [189]. The RAIN-701 trial is a preclinical model in which tarloxotinib was evaluated in patients with advanced non-small cell lung cancer (NSCLC) harboring EGFR Exon 20 insertions or HER2 activating mutations, as well as those with NRG1, EGFR, HER2, or HER4 gene fusions. In total, 23 patients were treated with tarloxotinib at 150 mg/m^2^ weekly. The overall disease control rate was 60%, with 22% of patients in the HER2 cohort achieving a partial response (PR). Most treatment-emergent adverse events were grade 1/2, including prolonged QTc (60.9%) and rash (43.5%). Notably, tarloxotinib showed antitumor activity in HER2-mutant NSCLC and was well tolerated, with low rates of severe EGFR-related toxicities such as rash and diarrhea. This study concluded that tarloxotinib has potential in this patient population, with ongoing trials to explore its efficacy further (NCT03805841) [197]. In the phase II study, among patients with cutaneous squamous cell carcinoma (CSCC), only 3% (1 out of 30) achieved a partial response, lasting 6.9 months, while 46% (14 out of 30) experienced progressive disease. The progression-free survival and overall survival curves were reported, though specific statistical values for PFS and OS were not provided. The most common adverse events were QTc prolongation (37%) and rash (20%), with 14 patients requiring dose reductions due to adverse events. This study did not meet its primary endpoint of a ≥5% response rate, and tarloxotinib’s efficacy was considered limited, with no significant survival benefit. Despite preclinical promise, the results did not show substantial clinical efficacy, and this study did not progress to the second stage [188].

### 5.2. Oxygenation Therapies

Oxygenation therapies aim to increase oxygen levels within the tumor microenvironment to enhance cancer cell sensitivity to conventional treatments [198]. Hyperbaric oxygen therapy (HBOT) involves breathing 100% oxygen at elevated atmospheric pressure in a hyperbaric chamber [199]. Protocols typically include 90–120 min sessions, administered one–three times daily, with 20–60 treatments at pressures of two to three atmospheres [200]. This increases dissolved oxygen in plasma, improving delivery to hypoxic tissues. HBOT is a safe and well-established method for rapidly increasing oxygen delivery to tissues, enhancing it by as much as 10- to 20-fold [201]. Since oxygen is vital for processes like immune defense, fibroblast activation, collagen deposition, angiogenesis, and epithelialization, there were concerns that the HBOT might promote tumor growth [199]. However, current evidence strongly suggests that HBOT does not enhance tumor progression and may even reduce primary tumor size [202].

Currently, HBOT is recognized in clinical practice as an effective strategy for managing radiation-induced injuries and other anticipated complications associated with cancer therapies [203]. Beyond its role in supportive care, increasing evidence suggests that HBOT may improve treatment efficacy in certain malignancies. Granowitz et al. showed that HBOT alone inhibited proliferation in both benign and malignant mammary epithelial cell lines [204]. Similarly, in a murine model of colorectal cancer, combining HBOT with 5-fluorouracil (5-FU) increased apoptosis in dysplastic crypts and resulted in improved treatment outcomes compared to chemotherapy alone [205]. In vitro, HBOT inhibited the growth of A549 lung cancer cells in a time-dependent manner and rapidly decreased p53 protein expression. This reduction was reversible by a proteasome degradation inhibitor, but not by an autophagy inhibitor, suggesting that HBOT mediates p53 downregulation via proteasomal degradation. Furthermore, in mouse xenograft models, HBOT enhanced tumor tissue angiogenesis, alleviated tumor hypoxia, and promoted tumor cell apoptosis by modulating the hypoxic tumor microenvironment, thereby suppressing lung cancer progression [206].

Hypoxic tumor regions are well known to resist radiation therapy. HBOT increases tissue oxygen tension, thereby enhancing radiotherapy efficacy [207]. In glioblastoma models, Chun-Man Yuen et al. demonstrated that while HBOT did not significantly affect overall tumor cell viability, it markedly reduced cancer stem cell formation and increased sensitivity to both temozolomide and radiotherapy in vitro and in vivo [208]. Additionally, Poff et al. proposed a safe metabolic cancer therapy combining a ketogenic diet, ketone supplementation, and HBOT. This multifaceted approach targets tumor glucose dependence and elevates oxidative stress, effectively inhibiting tumor growth and prolonging survival in animal models [209]. Notably, hematopoietic-derived cancer cells exhibit a lower oxidative stress threshold to HBOT. The induced apoptosis may be mediated by intracellular accumulation of H_2_O_2_ and (O_2_•^−^, alongside p38 MAPK phosphorylation [210].

HBOT also enhances nanomedicine delivery in solid tumors by reducing hypoxia and HIF-1α expression. This improves the efficacy of Doxil in hepatocellular carcinoma (HCC) H22 tumors and nanoparticle-based photodynamic therapy in triple-negative breast cancer (TNBC) 4T1 models [211]. At the molecular level, repeated HBOT exposure induces gene expression changes in glioma cell lines, correlating with improved survival. RNA microarray analyses identified 17 genes, including COL1A1, ADAMTS1, and PTBP3, as potential therapeutic targets and biomarkers, suggesting roles in personalized treatment. However, clinical validation is needed [212]. Furthermore, oxygen therapy enhances cytotoxic T lymphocyte and natural killer (NK) cell activity and proliferation, contributing to tumor cell elimination [213]. Synthetic oxygen carriers mimicking hemoglobin are also under investigation as alternative strategies to improve oxygen delivery to hypoxic tumor regions [213].

Research on HBOT in cancer treatment is complex due to the diversity of experimental designs and therapeutic protocols. To clarify the variability in tumor response to oxygenation, we summarized available data on the effects of HBO on cancer hallmarks, its influence on chemo- and radiotherapy, and the responses across different tumor types (Table 3).

### 5.3. HIF Signaling Inhibitors

Regarding HIF mediating cell response to hypoxia, many inhibitors of HIF signaling have been developed. Numerous components act through different mechanisms, for example decreasing HIF-1a mRNA expression, decreasing HIF-1a protein synthesis, decreasing HIF-1a stabilization, and decreasing HIF transcriptional activity, as shown in Table 3. Despite attempts, clinical translation of HIF inhibitors has not been fully successful to date [219]. Findings concerning key outcomes of clinical trials involving HIF signaling inhibitors are collected and summarized in Table 4.

#### 5.3.1. Inhibitors of HIF1 mRNA Expression

EZN-2208, a water-soluble PEGylated conjugate of the topoisomerase I inhibitor SN38 is an active metabolite of irinotecan that exerts anti-hypoxic effects primarily through the downregulation of HIF-1α mRNA expression. Several phase I clinical trials have demonstrated that EZN-2208 is generally safe and well-tolerated. In one study involving patients with neuroblastoma, EZN-2208 was administered at a dose of 24 mg/m^2,^ and no grade 4 or 5 adverse events have been reported [174,219,221]. The most commonly observed adverse effects included neutropenia, diarrhea, and leukopenia. Furthermore, a study by Jeong et al. reported that the combination of EZN-2208 with bevacizumab was clinically acceptable (prolonged disease stabilization in two patients) and resulted in reduced HIF-1α protein levels in tumor biopsy samples from five out of seven patients [222].

#### 5.3.2. Inhibitors of HIF-1 Synthesis

EZN-2968 is a locked nucleic acid (LNA) antisense oligodeoxynucleotide that exerts its inhibitory effect on HIF-1 by specifically blocking the translation of HIF-1α mRNA, thereby preventing synthesis of the HIF-1α protein [20,28]. In a study conducted by Jeong et al., administration of EZN-2968 at a dose of 18 mg/kg twice within a 6-week cycle resulted in disease stabilization in one patient with refractory solid tumors and a reduction in HIF-1α protein levels in tumor biopsies from two patients [224].

Topotecan, a topoisomerase I inhibitor, has also demonstrated potential as an inhibitor of HIF-1α [220,223]. In the referenced study, topotecan was administered at a daily dose of 1.2 mg/m^2^ and was found to be tolerable without inducing severe toxicity. Among 16 patients treated, 7 showed measurable responses, including decreased tumor perfusion in 4 patients and partial tumor response in 1 patient, as assessed by dynamic contrast-enhanced magnetic resonance imaging.

#### 5.3.3. Agents Reducing HIF-1 Stability

- Romidepsin

Romidepsin (FK228), a histone deacetylase (HDAC) inhibitor, exerts its effects in part by reducing the stabilization of HIF-1 [174,219]. Multiple phase I clinical trials have demonstrated that romidepsin is generally safe and well-tolerated. In a study by Gerber et al., romidepsin administered at 8 mg/m^2^ in combination with erlotinib showed promising activity in patients with advanced non-small cell lung cancer [273]. Similarly, another study reported that a 12 mg dose of romidepsin combined with high-dose chemotherapy was feasible and appropriate for phase II investigation in T-cell lymphoma [274]. A multicenter phase I/II study found that romidepsin at 14 mg/m^2^ in combination with tenalisib (800 mg) was tolerable and identified as the recommended phase II dose. No dose-limiting toxicities were observed, and adverse events were deemed manageable [226]. Additional evidence from a recent phase I trial in relapsed/refractory lymphomas demonstrated that romidepsin at 10 mg/m^2^ was well-tolerated and effective when combined with GEM, oxaliplatin, and dexamethasone, resulting in an overall response rate (ORR) of 60%. The most commonly reported adverse effects associated with romidepsin included thrombocytopenia, lymphopenia, and fatigue [227].

Romidepsin (10 mg) was also assessed in combination with duvelisib, and it has been observed that the regimen was safe, tolerable, and demonstrated substantial efficacy. The complete response rate (CRR) was 55% in the duvelisib + romidepsin arm, compared to 34% in the duvelisib + bortezomib group. Notably, this combination was associated with a lower incidence of grade 3/4 hepatotoxicity (14%, 8/59 patients) compared to a previously reported 40% (14/35 patients) with duvelisib monotherapy [228]. Despite promising results in early-phase trials, data on romidepsin’s efficacy remain inconclusive. A phase III study involving 421 patients with previously untreated peripheral T-cell lymphoma found no significant benefit when romidepsin was added to chemotherapy. Similarly, a phase II study showed no additional therapeutic advantage when romidepsin was combined with cyclophosphamide, doxorubicin, vincristine, and prednisone (CHOP) [274,275]. Conversely, a recent multicenter phase II trial by Ruan et al. reported that the combination of romidepsin and lenalidomide was as effective as conventional chemotherapy, and could serve as a viable alternative for patients ineligible for cytotoxic regimens [225].

- Vorinostat

Vorinostat, another representative of HDAC inhibitors, destabilizes HIF-1 by promoting the acetylation of its molecular chaperone, heat shock protein 90 (Hsp90), thereby impairing HIF-1 stability and function [20,28]. Preclinical and clinical studies suggest that vorinostat is generally well-tolerated. The most commonly reported adverse events are mainly typical and include hematologic toxicities (leukopenia, thrombocytopenia, and anemia), gastrointestinal symptoms (nausea, vomiting, diarrhea), hypophosphatemia, acute kidney injury, and hypothyroidism. A phase I clinical trial in patients with advanced prostate, renal, and urothelial carcinomas assessed the combination of pembrolizumab and vorinostat. The regimen was well-tolerated, with no dose-limiting toxicities (DLTs) observed, and a recommended phase II dose (RP2D) of 200 mg was established. However, clinical efficacy was limited and observed only in a subset of patients [229]. In a separate phase I trial by Godfrey et al., evaluating pembrolizumab combined with vorinostat (100–200 mg) in relapsed/refractory B-cell non-Hodgkin lymphoma, only one patient experienced a DLT at the 200 mg dose; thereby, RP2D was confirmed at 200 mg. The combination was particularly effective in PD-L1-high subtypes such as primary mediastinal B-cell lymphoma (PMB), suggesting that vorinostat may potentiate the efficacy of PD-1 inhibition [230]. Stitzlein et al. conducted a phase I study of vorinostat in combination with temsirolimus in patients with newly diagnosed or progressive diffuse intrinsic pontine glioma (DIPG). The regimen was well-tolerated, with only one patient experiencing grade 3 leukopenia. The maximum tolerated dose (MTD) for vorinostat was identified as 230 mg/m^2^ [231]. Vorinostat is FDA-approved for the treatment of cutaneous T-cell lymphoma; however, its efficacy in other malignancies remains uncertain. In a phase II study, vorinostat (400 mg) combined with hydroxychloroquine did not demonstrate clinical efficacy in patients with chemotherapy-refractory metastatic colorectal cancer, despite some observed immunomodulatory effects [232]. A phase III, double-blind, randomized trial in patients with advanced malignant pleural mesothelioma found no improvement in overall survival with vorinostat treatment compared to placebo [233]. Similarly, two other phase III randomized trials reported no therapeutic benefit of vorinostat. In the Myeloma XI study by Jenner et al., patients receiving 300 mg of vorinostat in combination with lenalidomide experienced a higher incidence of grade 3 and 4 hematologic toxicities (neutropenia, thrombocytopenia, and anemia) without improved outcomes [234]. Garcia-Manero et al. evaluated vorinostat added to standard IA (idarubicin and cytarabine) chemotherapy in acute myeloid leukemia (AML) and found no enhancement in overall survival, event-free survival, or response rates. Furthermore, the addition of vorinostat was associated with increased treatment-related toxicity [235].

- Panobinostat

Panobinostat (LBH589), a hydroxamic acid derivative that also belongs to HDAC inhibitors, disrupts the HSP90/HDAC6 complex, leading to the destabilization of HIF-1α [174,219]. Commonly reported adverse events associated with panobinostat include myelosuppression, gastrointestinal symptoms, fatigue, fever, peripheral edema, cough, and pruritus [237,238]. It has demonstrated clinical efficacy in the treatment of multiple myeloma, particularly when used in combination with other agents. In a randomized, double-blind, phase III trial, patients receiving 20 mg of panobinostat in combination with bortezomib and dexamethasone exhibited significantly prolonged progression-free survival compared to the control group (11.99 months vs. 8.08 months) [239]. These findings established 20 mg as a safe and effective dose in this setting. Similarly, a phase III study conducted by Richardson et al. confirmed the clinical benefit of adding panobinostat (20 mg) to bortezomib and dexamethasone, reinforcing its therapeutic potential in relapsed or refractory multiple myeloma [240]. Further supporting these results, Laubach et al. performed a phase II trial that validated the safety and efficacy of this combination regimen [236].

- Tanespimycin

Tanespimycin (17-AAG), a derivative of the antibiotic geldanamycin, is an inhibitor of Hsp90 and functions by destabilizing HIF-1α through disruption of its chaperone-mediated stabilization [174,219]. Multiple phase I clinical trials have demonstrated that tanespimycin is generally safe and well-tolerated at doses ranging from 150 to 500 mg/m^2^. In a phase I study, the recommended phase II dose (RP2D) for tanespimycin in combination with bortezomib was determined to be 200–250 mg/m^2^ administered twice weekly. Walker et al. identified the maximum tolerated dose (MTD) at 150 mg/m^2^ for the same combination, whereas Richardson et al. reported good tolerability at doses up to 340 mg/m^2^ [244,246,247]. Vaishampayan et al. suggested 400 mg/m^2^ as an appropriate dose for further evaluation, and Iyer et al. supported a dosage of 500 mg/m^2^ when used in combination with docetaxel [243,245]. The most frequently reported adverse events associated with tanespimycin were manageable and included fatigue, gastrointestinal disturbances, hematologic toxicities (thrombocytopenia, leukopenia), and peripheral sensory neuropathy. Despite its acceptable safety profile, the clinical efficacy of tanespimycin remains inconclusive. No significant clinical responses were observed in a study evaluating tanespimycin in metastatic, hormone-refractory prostate cancer [241]. Similarly, in a phase II trial, Hendrickson et al. reported limited therapeutic benefit in patients with advanced epithelial ovarian and primary peritoneal carcinomas [242]. However, a study investigating the combination of tanespimycin with sorafenib in patients with renal cell carcinoma and melanoma reported clinical benefit in approximately 70% of participants, suggesting potential utility in selected malignancies [245].

- Lonafarnib

Lonafarnib (SCH66336) is a farnesyltransferase inhibitor that contributes to the destabilization of HIF-1α, also by disrupting its interaction with Hsp90 [174,219]. Clinical data suggest that lonafarnib is generally well-tolerated across a range of dosing regimens and tumor types. In a study by Yust-Katz et al., lonafarnib, administered at a dose of 200 mg in combination with temozolomide, was associated with 6-month progression-free survival (PFS) in nearly 40% of patients with recurrent or temozolomide-refractory glioblastoma [248]. Daily administration of lonafarnib at 250 mg in patients with HER2-positive advanced breast cancer was shown to be safe and well-tolerated, with signs of antitumor activity observed in approximately 60% of participants [249]. A phase I multicenter trial established that lonafarnib at 150 mg daily was a safe dosage for further clinical evaluation. This study reported partial responses in two patients and disease stabilization in twelve others [250]. Similarly, Kim et al. found that lonafarnib at 100 mg twice daily in combination with paclitaxel was well-tolerated in patients with non-small cell lung cancer (NSCLC), with manageable adverse effects and clinical benefit observed in 14 patients (3 with partial responses and 11 with stable disease) [251]. However, other studies have reported limited or no clinical benefit from lonafarnib. Meier et al. evaluated the addition of lonafarnib (100 mg twice daily) to carboplatin and paclitaxel in advanced ovarian cancer and found no improvement in progression-free or overall survival. Similarly, the use of 200 mg of lonafarnib twice daily in patients with squamous cell carcinoma of the head and neck demonstrated good tolerability but no meaningful clinical efficacy [252].

- Belinostat

Belinostat (PXD-101) is a small-molecule pan-histone deacetylase (HDAC) inhibitor known to destabilize HIF-1. Clinical studies have demonstrated it is generally well-tolerated at doses ranging from 500 mg/m^2^ up to 2000 mg/m^2^. In a phase I study, Shafer et al. evaluated belinostat (1000 mg/m^2^) in combination with adavosertib in patients with relapsed or refractory malignancies. While the combination was found to be safe, no significant clinical benefits were observed [253]. The efficacy of belinostat in combination with 13-cis-retinoic acid (13-cRA) in patients with advanced solid tumors was assessed, confirming the tolerability of belinostat at doses of up to 2000 mg/m^2^/day [254]. Importantly, belinostat administered at 1000 mg/m^2^ demonstrated clinical efficacy in patients with relapsed or refractory peripheral T-cell lymphoma (PTCL), leading to improved overall response rates and subsequent regulatory approval for use as monotherapy in this indication [255,256,258]. Recent evidence also supports the potential role of belinostat in other hematologic malignancies. In a study by Maher et al., belinostat at 800–1000 mg/m^2^ combined with pevonedistat yielded clinical benefit in heavily pretreated patients with relapsed or refractory acute myeloid leukemia (AML), including one complete remission and disease stabilization in other patients [257]. Additionally, Herbaux et al. demonstrated that belinostat enhanced apoptosis and sensitized leukemic cells to venetoclax in T-cell prolymphocytic leukemia (T-PLL), suggesting potential for use in combination regimens [260]. In glioblastoma, belinostat has shown promise when added to standard chemoradiation therapy (radiotherapy and temozolomide). A phase I trial evaluating belinostat at doses of 500 mg/m^2^ for five consecutive days in combination with chemoradiotherapy reported improved median overall survival (~2.7 months longer) and enhanced 6-month progression-free survival compared to controls. The 500 mg/m^2^ dose was well-tolerated, whereas the 750 mg/m^2^ dose was associated with dose-limiting toxicities, including grade 4 thrombocytopenia and neutropenia [259].

- Chidamide

Chidamide (also known as tucidinostat), a selective inhibitor of HDAC1, 2, 3, and 10, has been the focus of numerous recent clinical trials, demonstrating both safety and therapeutic efficacy across various hematologic malignancies. In a phase II trial, Zou et al. reported that chidamide administered at 20 mg twice weekly was well-tolerated and exhibited promising anti-tumor activity when combined with rituximab, gemcitabine, and oxaliplatin (R-GemOx) in patients with relapsed or refractory diffuse large B-cell lymphoma (R/R DLBCL). Their study reported a high disease control rate (DCR) of 77.8%, with a median duration of response (DOR) of 11.6 months among responders [262]. Similarly, a phase II study evaluated chidamide at 30 mg twice weekly in combination with rituximab, gemcitabine, cisplatin, and dexamethasone, also in R/R DLBCL. The combination was well-tolerated and demonstrated an overall response rate (ORR) of 79.1%, including a complete response rate of 45.8% and a partial response rate of 33.3% [263]. Both studies reported hematologic toxicities, including anemia, lymphocytopenia, and thrombocytopenia, as the most common adverse events. Chidamide has also shown clinical benefit in the treatment of peripheral T-cell lymphoma (PTCL). In a phase II study, Wang et al. demonstrated that maintenance therapy with chidamide following first-line chemotherapy significantly improved both progression-free survival (PFS) and overall survival (OS) compared to observation alone. Notably, the survival outcomes with chidamide maintenance were comparable to those achieved with autologous stem cell transplantation (ASCT), suggesting its potential as a viable post-remission alternative in PTCL management [264]. In patients unable to tolerate conventional chemotherapy, Liang et al. reported that a combination of chidamide (30 mg twice weekly) with prednisone, cyclophosphamide, and thalidomide was effective in relapsed or refractory PTCL, with hematologic toxicities again being the most frequent adverse events [265]. Gao et al. proposed a recommended phase II dose (RP2D) of 30 mg twice weekly for chidamide based on a phase Ib/II study evaluating its use in combination with sintilimab (anti-PD-1 antibody) for relapsed/refractory extranodal natural killer/T-cell lymphoma (ENKTL), supporting its safety and efficacy in this rare subtype [266]. Furthermore, in the setting of hemophagocytic lymphohistiocytosis (HLH), Xu et al. investigated the addition of chidamide to etoposide and glucocorticosteroids. Their study observed an overall response in 13 of 17 patients, with manageable hematologic toxicities and very few grade 3/4 adverse events, reinforcing chidamide’s favorable safety profile in this context [267].

#### 5.3.4. Inhibitors of HIF-1 Transcriptional Activity

Suppressing the HIF-1α function as a transcription factor is also a promising area in the development of new therapeutic agents, attenuating hypoxia in cancer. Bortezomib is a proteasome inhibitor that suppresses transcriptional activity of HIF-1α by interfering with its degradation pathway and downstream signaling. Bortezomib is already used in the therapy of multiple myeloma in combination with cyclophosphamide and dexamethasone and as a therapy option for mantle cell lymphoma. Nonetheless, it is constantly being assessed for other indications. Clinical investigations have demonstrated its efficacy and tolerability across various malignancies, both as monotherapy and in combination regimens. Falchook et al. conducted a phase I trial evaluating the combination of bortezomib with bevacizumab in patients with advanced, refractory malignancies. Bortezomib was administered at an RP2D of 1.3 mg/m^2^ and was well-tolerated. Their study reported four partial responses and disease stabilization in 39 patients. Notably, post-treatment biopsies revealed decreased HIF-1α expression in patients who had positive baseline HIF-1α staining, confirming target engagement [268]. In a separate phase I study, Aubrey et al. demonstrated that bortezomib (1.3 mg/m^2^), in combination with lenalidomide, could be safely administered following donor lymphocyte infusion in patients with relapsed AML or myelodysplastic syndrome after allogeneic stem cell transplantation. Toxicities were manageable and primarily consisted of cytopenias during induction [272]. Fischer et al. evaluated bortezomib as part of a multi-agent regimen (rituximab, high-dose cytarabine, and dexamethasone) in relapsed/refractory mantle cell lymphoma (R/R MCL). The addition of bortezomib significantly improved the ORR and CRR compared to the control group [271]. Choi et al. investigated the efficacy of bortezomib combined with dexamethasone in patients with relapsed/refractory cutaneous T-cell lymphoma (CTCL). An overall response was observed in 13 of 29 patients (44.8%), demonstrating the regimen’s potential in this patient population [269]. Additionally, Zeng et al. reported clinical benefits from bortezomib in a cohort of patients with advanced intrahepatic cholangiocarcinoma harboring PTEN deficiencies. Seven patients experienced clinical improvement, suggesting that bortezomib may offer therapeutic value in this otherwise treatment-refractory cancer [270].

## 6. Summary and Conclusions

Tumor hypoxia plays a critical role in cancer progression by promoting treatment-resistant phenotypes, enhancing stem-like cell traits, increasing metastatic potential, and driving resistance to both radiotherapy and chemotherapy. Hypoxia impairs DNA repair mechanisms, particularly HR, by downregulating key genes such as RAD51, BRCA1, and FANCD2, as well as through microRNA regulation and epigenetic modifications. This disruption leads to increased genomic instability. The impact of hypoxia on NHEJ is more complex: while acute hypoxia may stimulate this pathway, chronic hypoxia generally suppresses it by decreasing levels of critical proteins like LIGIV, XRCC4, and PTEN. Although the inhibition of DNA repair under hypoxic conditions promotes genomic instability and mutagenesis, it simultaneously opens up new therapeutic avenues. Building on the proof of concept established by first-generation agents like tirapazamine, newer compounds such as PR-104, CP-506, evofosfamide, and tarloxotinib demonstrate enhanced hypoxia selectivity, varied cytotoxic mechanisms, and improved tolerability. Preclinical and early clinical studies have demonstrated efficacy across various tumor types, particularly when used in combination with chemotherapy, radiotherapy, or immunotherapy. These advances represent significant progress in targeting tumor hypoxia while minimizing systemic toxicity. However, successful clinical translation depends on the development of robust hypoxia biomarkers, better patient stratification, and a deeper understanding of resistance mechanisms. HIF-1 inhibitors show therapeutic potential, but most clinical studies to date have involved small patient cohorts, underscoring the need for larger, well-designed clinical trials to validate their efficacy and safety.

## Figures and Tables

**Table 1:** Key Biomarkers Reflecting Tumor Hypoxia: Biological Insights and Clinical Relevance.

Biomarker	Biological Function	Detection Method	Clinical Relevance	Refs.
HIF-1α	A master regulator of cellular response to low oxygen; accumulates under hypoxia and binds hypoxia-response elements (HRE) on DNA to promote cell survival(targets include CA-IX, LOX, and GLUT-1).	Immunohistochemistry (IHC), Western blot (WB), qRT-PCR, ELISA, ChI	A meta-analysis involving 5177 patients demonstrated that high HIF-1α expression is associated with poorer survival and more aggressive breast cancer features, including overall survival (OS), disease-free survival (DFS), tumor stage (TNM), and estrogen receptor (ER) status; increased expression predicts poor prognosis in ovarian cancer and osteosarcoma.	[109,110,111,112]
Carbonic anhydrase IX (CAIX)	Regulates pH in hypoxic tumor cells by converting CO_2_ to bicarbonate and protons, supporting survival in acidic conditions.	IHC, ELISA, PET imaging with CAIX-targeted radiotracers	A prospective analysis involving high-risk non-metastatic ccRCC patients confirmed CAIX expression as a statistically significant prognostic biomarker for DFS and OS. High plasma CAIX levels serve as an independent prognostic biomarker in patients with non-small cell lung cancer (NSCLC), particularly in early stages (I and II).	[113,114]
Vascular endothelial growth factor (VEGF)	Key regulator of angiogenesis, promoting blood vessel formation to supply oxygen and nutrients, and modulating the tumor immune microenvironment.	ELISA (serum/plasma), IHC, qRT-PCR	Serum VEGF-A helps identify NSCLC patients benefiting from bevacizumab; higher hypoxia-related VEGF scores in necrotic breast tumors, especially basal-like subtype VEGF levels are affected by various factors besides hypoxia, which results in a variable correlation with direct measures of tumor oxygenation like pimonidazole.	[115,116,117]
Glucose transporter 1 (GLUT-1)	Glucose transporter is upregulated by HIF-1α under hypoxia to increase glucose uptake for anaerobic metabolism	IHC, WB, qRT-PCR, ELISA	Serum levels rise after radiotherapy in glioma, indicating tumor metabolic adaptation; overexpression is linked to radioresistance in breast cancer. High GLUT-1 correlates with poor prognosis in head and neck, lung, and pancreatic cancers, serving as a potential prognostic and therapeutic biomarker.	[118,119,120]
Lactate dehydrogenase (LDH)	Critical metabolic enzyme involved in glucose and glutamine metabolism, tumor pH regulation, and TCA cycle activity.	ELISA, serum biochemical assays	Elevated salivary LDH is found in HNC and OPMD compared to controls; serum LDH is prognostic in several cancers, including colorectal and prostate cancers. Activity correlates with aggressive breast cancer features.	[121,122]
Lysyl oxidase (LOX)	Involved in ECM remodeling and hypoxia-induced repression of E-cadherin via HIF-1.	IHC, qRT-PCR (tissue and blood samples)	LOX and HIF-1α expression increase with lymph node metastasis and tumor invasion, correlating with gastric cancer progression and poor prognosis; additionally, LOX is linked to patient survival, glioma cell differentiation, tumor recurrence, and may serve as a prognostic biomarker and therapeutic target for glioblastoma.	[123,124,125]
miR-210	Regulates cellular metabolism under hypoxia by shifting energy production from oxidative phosphorylation to glycolysis; suppresses mitochondrial enzymes and increases ROS to support survival.	qRT-PCR (from plasma, serum, or tissue), microarray, next-generation sequencing (NGS)	MicroRNA-210 may serve as a biomarker for NSCLC detection; its high expression predicts poor prognosis in breast cancer and reflects hypoxia in bladder cancer, correlating with other markers, while microarray analyses identify it as a key hypoxia-induced miRNA across breast, head and neck, lung, colon, and kidney cancer cell lines.	[126,127,128,129]
Pimonidazole (PIMO)	Exogenous hypoxia marker (2-nitroimidazole) binds covalently to cellular macromolecules when oxygen levels drop below 1.3%.	IHC after administration as hypoxia marker	Oral PIMO revealed aggressive clinico-pathological features in localized prostate cancer Imaging-guided biopsies from 52 patients (43 given pimonidazole) showed aggressive hypoxia-driven prostate cancer phenotype, validated in two cohorts. Digital image analysis shows distinct differences between HIF-1α and PIMO as hypoxia biomarkers, indicating coexistence of different hypoxia forms in laryngeal cancer.	[130,131,132]

**Table 2:** Hypoxia-activated prodrugs in cancer therapies.

Hypoxia-Activated Prodrug (HAP)	Mechanism of Action	Key Outcomes	References
Tirapazamine	reduced to cytotoxic radical species in a hypoxic environment	improves outcome in non-operative hepatocellular cancer combined with transarterial embolization	[175,176,177]
PR-104	converted to PR-104A, activated under hypoxia via one-electron reduction by cytochrome P450 oxidoreductase, generating cytotoxic metabolites	decreased tumor burden and prolonged survival in preclinical models of ALL, T-ALL, and AML, associated with disease response in a Phase I/II clinical trial	[178,179,180]
CP-506	undergoes metabolic activation in hypoxic conditions and targets negatively charged guanine bases, disrupting DNA replication and leading to the death of hypoxic tumor cells	improvement in patients with hypopharyngeal squamous cell carcinoma	[173,181]
Evofosfamide (TH-302)	releases a DNA-crosslinking bromo-isophosphoramide mustard (Br-IPM) under low-oxygen conditions	promising results in trials with pancreatic cancer, prostate cancer, and melanoma	[182,183,184,185,186,187]
Tarloxotinib	activated only in low-oxygen conditions, releasing tarloxotinib-TKI, which irreversibly inhibits the pan-HER family (EGFR, HER2, HER4) by binding to conserved cysteine residues, blocking ERK and AKT pathways to suppress cell proliferation and survival	may help avoid bone marrow suppression, a common issue with cytotoxic HAPs that could limit their effectiveness when paired with ICIs	[188,189]

**Table 3:** Summary of the Biological Effects of Hyperbaric Oxygen Therapy (HBO).

Biological Aspect	Effect of HBO	Key Outcomes	References
Apoptosis	Apoptosis induction in tumor cells	Activation of pro-apoptotic MAPK and inhibition of anti-apoptotic ERK in hematopoietic cells; Induction of apoptosis in osteosarcoma cells.	[210,214]
Tumor Angiogenesis	May inhibit neovascularization, but remains controversial	Reduced tumor peripheral vessel diameter and density in breast cancer and glioma models; Improved angiogenesis without increasing tumor growth in lung cancer xenograft models; Downregulation of proangiogenic genes (*VEGFα*, *VEGFβ*, *FGFM, PDGF*, *TGFα*) in rat adenocarcinoma.	[206,215,216]
Metastatic Potential	Does not enhance metastasis, may reduce tumor invasiveness by promoting a MET-like phenotype	No metastasis induced by HBO was observed in vivo in mouse models of squamous cell carcinoma and head and neck cancer; HBO promotes MET in DMBA-induced breast tumors, leading to less aggressive phenotype.	[202,216]
Chemotherapeutic Efficacy	Increases tumor perfusion and cellular sensitivity, enhances drug delivery to hypoxic tumor regions	Increased vascularization in large tumors (e.g., epithelial ovarian cancer), improving chemotherapy response; Increased GBM sensitivity to temozolomide; Slowed tumor growth and increased chemo efficacy in rat mammary tumors.	[217,218]
Radiotherapy Efficacy	Therapeutic agent for late radiation injuries; radiosensitizer enhancing radiotherapy effect	Study on 320 cervical cancer patients treated with radiotherapy and HBO showed significant improvement in local control and survival in the HBO group; Shown to reduce late radiation-induced injuries in the head and neck region, bones, prostate, and bladder.	[164,207]

**Table 4:** Clinical trials of HIF-1α inhibitors in cancer therapies.

HIF-1 Inhibitor	Mechanism of Action	Key Outcomes	References
EZN-2208	Decreasing HIF-1α mRNA expression	- Shown to be safe and well-tolerated in a dose of 12–30 mg/m^2^ in young adults with solid tumors; - Its addition to bevacizumab resulted in decreased levels of HIF-1α protein levels and prolonged disease stabilization.	[174,219,220,221,222]
Topotecan	Decreasing HIF-1α mRNA translation	Decreased HIF-1α expression in advanced solid tumors.	[223]
EZN-2968		- Decreased HIF-1 levels in patients with refractory solid tumors; - Prolonged disease stability.	[174,219,220,224]
Romidepsin (FK228)	Decreasing HIF-1α stabilization	- Safe and well-tolerable in dosage of 8–14 mg/m^2;^ - Inconclusive efficacy results.	[174,219,220,225,226,227,228]
Vorinostat		- Good tolerability of 100–400 mg doses; - Approved in therapy for cutaneous T-cell lymphoma, but failed to prove its efficacy in other cancer therapies.	[174,219,220,229,230,231,232,233,234,235]
Panobinostat (LBH589)		- A total of 20 mg panobinostat increased efficacy of multiple myeloma therapy.	[174,219,220,236,237,238,239,240]
Tanespimycin (17-AAG)		- Safe and well-tolerated at dosage 150–500 mg/m^2;^ - Unclear data concerning efficacy.	[174,219,220,241,242,243,244,245,246,247]
Lonafarnib (SCH66336)		- Well-tolerated in a dose of 150–200 mg twice a day; - Some promising data in the literature; however, further investigation is required.	[174,219,248,249,250,251,252]
Belinostat		- Effective in treatment of relapsed/refractory peripheral T-cell lymphoma; - Its addition was shown beneficial in relapsed/refractory acute leukemia, T-cell prolymphocytic leukemia, and glioblastoma.	[174,219,253,254,255,256,257,258,259,260]]
Chidamide		- Dose 20–30 mg 2x/week is well-tolerable; - Showed benefits in therapy of lymphoma and hemophagocytic lymphohistiocytosis.	[174,219,261,262,263,264,265,266,267]
Bortezomib	Decreasing HIF-1α transcriptional activity	- Safe and well-tolerated in a dose 1.3 mg/m^2^; - Effective in treatment of renal cell carcinoma, lymphoma, and intrahepatic cholangiocarcinoma.	[174,219,268,269,270,271,272]

## Data Availability

Not applicable.

## Abbreviations

The following abbreviations are used in this manuscript:

•OH	hydroxyl radicals
13-cRA	13-cis-retinoic acid
5-FU	5-fluorouracil
ABC	ATP-binding cassette
AMl	acute myeloid leukemia
ASCT	autologous stem cell transplantation
ATM	ataxia–telangiectasia-mutated kinase
ATMIN	ATM interactor
ATR	ataxia telangiectasia and Rad3-related kinase
ATRIP	ATR-interacting protein
BCRP	breast cancer resistance protein
Br-IPM	bromo-isophosphoramide mustard
CAFs	cancer-associated fibroblasts
CAIX	carbonic anhydrase IX
CHOP	cyclophosphamide, doxorubicin, vincristine, and prednisone
CR	complete response
CRi	incomplete count recovery
CRR	complete response rate
CSCC	cutaneous squamous-cell carcinoma
CSCs	cancer stem cells
CTCL	cutaneous T-cell lymphoma
DCR	disease control rate
DIPG	diffuse intrinsic pontine glioma
DLT	dose-limiting toxicity
DNA-PKcs	DNA-dependent protein kinase catalytic subunit
dNTP	deoxyribonucleotide
DOR	duration of response
DOX	doxorubicin
EGFR	epidermal growth factor receptor
EMT	epithelial–mesenchymal transition
EMT-TFs	EMT-related transcription factors
ENKTL	relapsed/refractory extranodal natural killer/T-cell lymphoma
EXO1	nuclease, exonuclease 1
FBP	fructose bisphosphatase
GEM	gemcitabine
H_2_O_2_	hydrogen peroxide
H3K4	fourth lysine residue on DNA packaging protein histone
HAPs	hypoxia-activated prodrugs
HBOT	hyperbaric oxygen therapy
HCC	hepatocellular carcinoma
HDAC	histone deacetylase
HIF	hypoxia-inducible factors
HK2	hexokinase 2
HLH	hemophagocytic lymphohistiocytosis
HNSCC	head and neck squamous cell carcinoma
HR	homologous recombination
HREs	hypoxia-response elements
ICI	immune checkpoint inhibitor
ITH	intratumoral heterogeneity
KDM	histone lysine demethylases
LOX	lysyl oxidase
MDSCs	myeloid-derived suppressor cells
MMP-9	matrix metalloproteinase-9
MRP1	multidrug resistance related protein 1
MTD	maximum tolerated dose
NHEJ	non-homologous end joining
NK	natural killer
NSCLC	non-small cell lung cancer
O_2_•^−^	oxygen superoxide
OER	Oxygen Enhancement Ratio
ORR	overall response rate
PARP-1	poly (ADP-ribose) polymerase-1
PDAC	pancreatic ductal adenocarcinoma
PFS	progression-free survival
P-gp	P-glycoprotein
PHDs	prolyl hydroxylases
PKM2	pyruvate kinase M2
PMB	primary mediastinal B-cell lymphoma
PR	partial response
PTCL	peripheral T-cell lymphoma
pVHL	von Hippel–Lindau tumor suppressor protein
R/R DLBCL	relapsed or refractory diffuse large B-cell lymphoma
R/R MCL	relapsed/refractory mantle cell lymphoma
R-GemOx	rituximab, gemcitabine, oxaliplatin
ROS	reactive oxygen species
RP2D	recommended phase II dose
RPA	replication protein A
SD	stable disease
SMO	smoothened
ssDNA	single-stranded DNA
TACE	trans-arterial chemoembolization
TAE	transarterial embolization
TAM	tumor-associated macrophage
TCA	tricarboxylic acid
TCR	T-cell receptor
TET	ten–eleven translocation
TGF-β	transforming growth factor β
TGI	tumor growth inhibition
TME	tumor microenvironment
TNBC	triple-negative breast cancer
T-PLL	T-cell prolymphocytic leukemia
VEGF	vascular endothelial growth factor
VGPR	very good partial responses
XPA	XP-A cells
XRCC4-LIGIV	DNA repair protein XRCC4 complex with ligase IV
ZEB1/2	zinc finger E-box binding homeobox 1/2

## References

[1] Bigos K.J., Quiles C.G., Lunj S., Smith D.J., Krause M., Troost E.G., West C.M., Hoskin P., Choudhury A. (2024). Tumour Response to Hypoxia: Understanding the Hypoxic Tumour Microenvironment to Improve Treatment Outcome in Solid Tumours. Front. Oncol..

[2] Bristow R.G., Hill R.P. (2008). Hypoxia, DNA Repair and Genetic Instability. Nat. Rev. Cancer.

[3] Vaupel P., Mayer A. (2007). Hypoxia in Cancer: Significance and Impact on Clinical Outcome. Cancer Metastasis Rev..

[4] Godet I., Shin Y.J., Ju J.A., Ye I.C., Wang G., Gilkes D.M. (2019). Fate-Mapping Post-Hypoxic Tumor Cells Reveals a ROS-Resistant Phenotype That Promotes Metastasis. Nat. Commun..

[5] Shi R., Liao C., Zhang Q. (2021). Hypoxia-Driven Effects in Cancer: Characterization, Mechanisms, and Therapeutic Implications. Cells.

[6] Al Tameemi W., Dale T.P., Al-Jumaily R.M.K., Forsyth N.R. (2019). Hypoxia-Modified Cancer Cell Metabolism. Front. Cell Dev. Biol..

[7] Barbeau L.M.O. (2024). Harnessing Autophagy in Cancer. Doctoral Thesis.

[8] Rahane D., Dhingra T., Chalavady G., Datta A., Ghosh B., Rana N., Borah A., Saraf S., Bhattacharya P. (2024). Hypoxia and Its Effect on the Cellular System. Cell Biochem. Funct..

[9] Chen N., Chen X., Huang R., Zeng H., Gong J., Meng W., Lu Y., Zhao F., Wang L., Zhou Q. (2009). BCL-XL Is a Target Gene Regulated by Hypoxia-Inducible Factor-1α. J. Biol. Chem..

[10] Choudhry H., Harris A.L. (2018). Advances in Hypoxia-Inducible Factor Biology. Cell Metab..

[11] Rocha H.L., Godet I., Kurtoglu F., Metzcar J., Konstantinopoulos K., Bhoyar S., Gilkes D.M., Macklin P. (2021). A Persistent Invasive Phenotype in Post-Hypoxic Tumor Cells Is Revealed by Fate Mapping and Computational Modeling. iScience.

[12] Emami Nejad A., Najafgholian S., Rostami A., Sistani A., Shojaeifar S., Esparvarinha M., Nedaeinia R., Haghjooy Javanmard S., Taherian M., Ahmadlou M. (2021). The Role of Hypoxia in the Tumor Microenvironment and Development of Cancer Stem Cell: A Novel Approach to Developing Treatment. Cancer Cell Int..

[13] Vallabhajosula S., Solnes L., Vallabhajosula B. (2011). A Broad Overview of Positron Emission Tomography Radiopharmaceuticals and Clinical Applications: What Is New?. Semin. Nucl. Med..

[14] Wozny A.-S., Alphonse G., Cassard A., Malésys C., Louati S., Beuve M., Lalle P., Ardail D., Nakajima T., Rodriguez-Lafrasse C. (2020). Impact of Hypoxia on the Double-Strand Break Repair after Photon and Carbon Ion Irradiation of Radioresistant HNSCC Cells. Sci. Rep..

[15] Scanlon S.E., Glazer P.M. (2015). Multifaceted Control of DNA Repair Pathways by the Hypoxic Tumor Microenvironment. DNA Repair.

[16] Muz B., de la Puente P., Azab F., Azab A.K. (2015). The Role of Hypoxia in Cancer Progression, Angiogenesis, Metastasis, and Resistance to Therapy. Hypoxia.

[17] Qian J., Rankin E.B. (2019). Hypoxia-Induced Phenotypes That Mediate Tumor Heterogeneity. Adv. Exp. Med. Biol..

[18] Vitale I., Shema E., Loi S., Galluzzi L. (2021). Intratumoral Heterogeneity in Cancer Progression and Response to Immunotherapy. Nat. Med..

[19] Watson I.R., Takahashi K., Futreal P.A., Chin L. (2013). Emerging Patterns of Somatic Mutations in Cancer. Nat. Rev. Genet..

[20] Ramón y Cajal S., Sesé M., Capdevila C., Aasen T., De Mattos-Arruda L., Diaz-Cano S.J., Hernández-Losa J., Castellví J. (2020). Clinical Implications of Intratumor Heterogeneity: Challenges and Opportunities. J. Mol. Med..

[21] Marusyk A., Janiszewska M., Polyak K. (2020). Intratumor Heterogeneity: The Rosetta Stone of Therapy Resistance. Cancer Cell.

[22] Thompson L.L., Jeusset L.M.-P., Lepage C.C., McManus K.J. (2017). Evolving Therapeutic Strategies to Exploit Chromosome Instability in Cancer. Cancers.

[23] Rath S., Chakraborty D., Pradhan J., Imran Khan M., Dandapat J. (2022). Epigenomic Interplay in Tumor Heterogeneity: Potential of Epidrugs as Adjunct Therapy. Cytokine.

[24] Guo M., Peng Y., Gao A., Du C., Herman J.G. (2019). Epigenetic Heterogeneity in Cancer. Biomark. Res..

[25] Meir Z., Mukamel Z., Chomsky E., Lifshitz A., Tanay A. (2020). Single-Cell Analysis of Clonal Maintenance of Transcriptional and Epigenetic States in Cancer Cells. Nat. Genet..

[26] Dietz S., Lifshitz A., Kazdal D., Harms A., Endris V., Winter H., Stenzinger A., Warth A., Sill M., Tanay A. (2019). Global DNA Methylation Reflects Spatial Heterogeneity and Molecular Evolution of Lung Adenocarcinomas. Int. J. Cancer.

[27] Runa F., Hamalian S., Meade K., Shisgal P., Gray P.C., Kelber J.A. (2017). Tumor Microenvironment Heterogeneity: Challenges and Opportunities. Curr. Mol. Biol. Rep..

[28] Jia Q., Wang A., Yuan Y., Zhu B., Long H. (2022). Heterogeneity of the Tumor Immune Microenvironment and Its Clinical Relevance. Exp. Hematol. Onco.l.

[29] Ge R., Wang Z., Cheng L. (2022). Tumor Microenvironment Heterogeneity an Important Mediator of Prostate Cancer Progression and Therapeutic Resistance. NPJ Precis. Oncol..

[30] Murphy K.J., Chambers C.R., Herrmann D., Timpson P., Pereira B.A. (2021). Dynamic Stromal Alterations Influence Tumor-Stroma Crosstalk to Promote Pancreatic Cancer and Treatment Resistance. Cancers.

[31] Wahl G.M., Spike B.T. (2017). Cell State Plasticity, Stem Cells, EMT, and the Generation of Intra-Tumoral Heterogeneity. NPJ Breast Cancer.

[32] Danhier P., Bański P., Payen V.L., Grasso D., Ippolito L., Sonveaux P., Porporato P.E. (2017). Cancer Metabolism in Space and Time: Beyond the Warburg Effect. Biochim. Biophys. Acta (BBA) Bioenerg..

[33] Jacquemin V., Antoine M., Dom G., Detours V., Maenhaut C., Dumont J.E. (2022). Dynamic Cancer Cell Heterogeneity: Diagnostic and Therapeutic Implications. Cancers.

[34] Strickaert A., Saiselet M., Dom G., De Deken X., Dumont J.E., Feron O., Sonveaux P., Maenhaut C. (2017). Cancer Heterogeneity Is Not Compatible with One Unique Cancer Cell Metabolic Map. Oncogene.

[35] Kim J., DeBerardinis R.J. (2019). Mechanisms and Implications of Metabolic Heterogeneity in Cancer. Cell Metab..

[36] Pandkar M.R., Dhamdhere S.G., Shukla S. (2021). Oxygen Gradient and Tumor Heterogeneity: The Chronicle of a Toxic Relationship. Biochim. Biophys. Acta (BBA) Rev. Cancer.

[37] Hompland T., Fjeldbo C.S., Lyng H. (2021). Tumor Hypoxia as a Barrier in Cancer Therapy: Why Levels Matter. Cancers.

[38] Magagnin M.G., Koritzinsky M., Wouters B.G. (2006). Patterns of Tumor Oxygenation and Their Influence on the Cellular Hypoxic Response and Hypoxia-Directed Therapies. Drug Resist. Updates.

[39] Michiels C., Tellier C., Feron O. (2016). Cycling Hypoxia: A Key Feature of the Tumor Microenvironment. Biochim. Biophys. Acta (BBA) Rev. Cancer.

[40] Chen Z., Han F., Du Y., Shi H., Zhou W. (2023). Hypoxic Microenvironment in Cancer: Molecular Mechanisms and Therapeutic Interventions. Signal Transduct. Target. Ther..

[41] Venkatesh G.H., Chakraborti S. (2022). Hypoxic Stress Perturb DNA Repair Mechanisms Leading to Genetic Instability. Handbook of Oxidative Stress in Cancer: Mechanistic Aspects.

[42] de Sá Junior P.L., Câmara D.A.D., Porcacchia A.S., Fonseca P.M.M., Jorge S.D., Araldi R.P., Ferreira A.K. (2017). The Roles of ROS in Cancer Heterogeneity and Therapy. Oxid. Med. Cell. Longev..

[43] Eales K.L., Hollinshead K.E.R., Tennant D.A. (2016). Hypoxia and Metabolic Adaptation of Cancer Cells. Oncogenesis.

[44] Abd G.M., Laird M.C., Ku J.C., Li Y. (2023). Hypoxia-Induced Cancer Cell Reprogramming: A Review on How Cancer Stem Cells Arise. Front. Oncol..

[45] Lehuédé C., Dupuy F., Rabinovitch R., Jones R.G., Siegel P.M. (2016). Metabolic Plasticity as a Determinant of Tumor Growth and Metastasis. Cancer Res..

[46] Špaková I., Rabajdová M., Mičková H., Graier W.F., Mareková M. (2021). Effect of Hypoxia Factors Gene Silencing on ROS Production and Metabolic Status of A375 Malignant Melanoma Cells. Sci. Rep..

[47] Terry S., Engelsen A.S.T., Buart S., Elsayed W.S., Venkatesh G.H., Chouaib S. (2020). Hypoxia-Driven Intratumor Heterogeneity and Immune Evasion. Cancer Lett..

[48] Simon M.C., Keith B. (2008). The Role of Oxygen Availability in Embryonic Development and Stem Cell Function. Nat. Rev. Mol. Cell. Biol..

[49] Hajizadeh F., Okoye I., Esmaily M., Ghasemi Chaleshtari M., Masjedi A., Azizi G., Irandoust M., Ghalamfarsa G., Jadidi-Niaragh F. (2019). Hypoxia Inducible Factors in the Tumor Microenvironment as Therapeutic Targets of Cancer Stem Cells. Life Sci..

[50] Wang P., Gong S., Liao B., Pan J., Wang J., Zou D., Zhao L., Xiong S., Deng Y., Yan Q. (2022). HIF1α/HIF2α Induces Glioma Cell Dedifferentiation into Cancer Stem Cells through Sox2 under Hypoxic Conditions. J. Cancer.

[51] Zhang Q., Han Z., Zhu Y., Chen J., Li W. (2020). Role of Hypoxia Inducible Factor-1 in Cancer Stem Cells (Review). Mol. Med. Rep..

[52] Onishi H., Nakamura K., Yanai K., Nagai S., Nakayama K., Oyama Y., Fujimura A., Ozono K., Yamasaki A. (2022). Cancer Therapy That Targets the Hedgehog Signaling Pathway Considering the Cancer Microenvironment (Review). Oncol. Rep..

[53] Kanwal R., Esposito J.E., Jawed B., Zakir S.K., Pulcini R., Martinotti R., Botteghi M., Gaudio F., Martinotti S., Toniato E. (2025). Exploring the Role of Epithelial–Mesenchymal Transcriptional Factors Involved in Hematological Malignancy and Solid Tumors: A Systematic Review. Cancers.

[54] Mrozik K.M., Blaschuk O.W., Cheong C.M., Zannettino A.C.W., Vandyke K. (2018). N-Cadherin in Cancer Metastasis, Its Emerging Role in Haematological Malignancies and Potential as a Therapeutic Target in Cancer. BMC Cancer.

[55] Zhang L., Huang G., Li X., Zhang Y., Jiang Y., Shen J., Liu J., Wang Q., Zhu J., Feng X. (2013). Hypoxia Induces Epithelial-Mesenchymal Transition via Activation of SNAI1 by Hypoxia-Inducible Factor-1α in Hepatocellular Carcinoma. BMC Cancer.

[56] Zheng H., Kang Y. (2014). Multilayer Control of the EMT Master Regulators. Oncogene.

[57] Tam S.Y., Wu V.W.C., Law H.K.W. (2020). Hypoxia-Induced Epithelial-Mesenchymal Transition in Cancers: HIF-1α and Beyond. Front. Oncol..

[58] Shen Z., Yu N., Zhang Y., Jia M., Sun Y., Li Y., Zhao L. (2024). The Potential Roles of HIF-1α in Epithelial-Mesenchymal Transition and Ferroptosis in Tumor Cells. Cell Signal..

[59] Yang J., Zhang X., Zhang Y., Zhu D., Zhang L., Li Y., Zhu Y., Li D., Zhou J. (2016). HIF-2α Promotes Epithelial-Mesenchymal Transition through Regulating Twist2 Binding to the Promoter of E-Cadherin in Pancreatic Cancer. J. Exp. Clin. Cancer Res..

[60] Belisario D.C., Kopecka J., Pasino M., Akman M., De Smaele E., Donadelli M., Riganti C. (2020). Hypoxia Dictates Metabolic Rewiring of Tumors: Implications for Chemoresistance. Cells.

[61] Lu W., Kang Y. (2019). Epithelial-Mesenchymal Plasticity in Cancer Progression and Metastasis. Dev. Cell.

[62] Tirpe A.A., Gulei D., Ciortea S.M., Crivii C., Berindan-Neagoe I. (2019). Hypoxia: Overview on Hypoxia-Mediated Mechanisms with a Focus on the Role of HIF Genes. Int. J. Mol. Sci..

[63] Song Y., Zheng S., Wang J., Long H., Fang L., Wang G., Li Z., Que T., Liu Y., Li Y. (2017). Hypoxia-Induced PLOD2 Promotes Proliferation, Migration and Invasion via PI3K/Akt Signaling in Glioma. Oncotarget.

[64] Lei Y., Chen L., Zhang G., Shan A., Ye C., Liang B., Sun J., Liao X., Zhu C., Chen Y. (2020). MicroRNAs Target the Wnt/Β catenin Signaling Pathway to Regulate Epithelial mesenchymal Transition in Cancer (Review). Oncol. Rep..

[65] Zhang Q., Bai X., Chen W., Ma T., Hu Q., Liang C., Xie S., Chen C., Hu L., Xu S. (2013). Wnt/β-Catenin Signaling Enhances Hypoxia-Induced Epithelial–Mesenchymal Transition in Hepatocellular Carcinoma via Crosstalk with Hif-1α Signaling. Carcinogenesis.

[66] De Francesco E.M., Maggiolini M., Musti A.M. (2018). Crosstalk between Notch, HIF-1α and GPER in Breast Cancer EMT. Int. J. Mol. Sci..

[67] Guo M., Niu Y., Xie M., Liu X., Li X. (2023). Notch Signaling, Hypoxia, and Cancer. Front. Oncol..

[68] Bao B., Azmi A.S., Ali S., Ahmad A., Li Y., Banerjee S., Kong D., Sarkar F.H. (2012). The Biological Kinship of Hypoxia with CSC and EMT and Their Relationship with Deregulated Expression of MiRNAs and Tumor Aggressiveness. Biochim. Biophys. Acta (BBA) Rev. Cancer.

[69] Fluegen G., Avivar-Valderas A., Wang Y., Padgen M.R., Williams J.K., Nobre A.R., Calvo V., Cheung J.F., Bravo-Cordero J.J., Entenberg D. (2017). Phenotypic Heterogeneity of Disseminated Tumour Cells Is Preset by Primary Tumour Hypoxic Microenvironments. Nat. Cell Biol..

[70] Pribluda A., de la Cruz C.C., Jackson E.L. (2015). Intratumoral Heterogeneity: From Diversity Comes Resistance. Clin. Cancer Res..

[71] Januškevičienė I., Petrikaitė V. (2019). Heterogeneity of Breast Cancer: The Importance of Interaction between Different Tumor Cell Populations. Life Sci..

[72] Kiwerska K., Szyfter K. (2019). DNA Repair in Cancer Initiation, Progression, and Therapy—A Double-Edged Sword. J. Appl. Genet..

[73] Zhou J., Albert Zhou X., Zhang N., Wang J. (2020). Evolving Insights: How DNA Repair Pathways Impact Cancer Evolution. Cancer Biol. Med..

[74] Shah M.A.F., Ud Din S., Shah A.A. Analysis of Machine Learning Techniques for Detection Framework for DNA Repair Genes to Help Diagnose Cancer: A Systematic Literature Review. Proceedings of the 2021 International Conference on Innovative Computing (ICIC).

[75] Hassan Venkatesh G., Bravo P., Shaaban Moustafa Elsayed W., Amirtharaj F., Wojtas B., Abou Khouzam R., Hussein Nawafleh H., Mallya S., Satyamoorthy K., Dessen P. (2020). Hypoxia Increases Mutational Load of Breast Cancer Cells through Frameshift Mutations. Oncoimmunology.

[76] Kaplan A.R., Glazer P.M. (2020). Impact of Hypoxia on DNA Repair and Genome Integrity. Mutagenesis.

[77] Scanlon S.E., Hegan D.C., Sulkowski P.L., Glazer P.M. (2018). Suppression of Homology-Dependent DNA Double-Strand Break Repair Induces PARP Inhibitor Sensitivity in VHL-Deficient Human Renal Cell Carcinoma. Oncotarget.

[78] Tang M., Bolderson E., O’Byrne K.J., Richard D.J. (2021). Tumor Hypoxia Drives Genomic Instability. Front. Cell Dev. Biol..

[79] Begg K., Tavassoli M. (2020). Inside the Hypoxic Tumour: Reprogramming of the DDR and Radioresistance. Cell Death Discov..

[80] Weterings E., Chen D.J. (2008). The Endless Tale of Non-Homologous End-Joining. Cell Res..

[81] Prabhu K.S., Kuttikrishnan S., Ahmad N., Habeeba U., Mariyam Z., Suleman M., Bhat A.A., Uddin S. (2024). H2AX: A Key Player in DNA Damage Response and a Promising Target for Cancer Therapy. Biomed. Pharmacother..

[82] Olcina M.M., Grand R.J., Hammond E.M. (2014). ATM Activation in Hypoxia-Causes and Consequences. Mol. Cell. Oncol..

[83] Paull T.T. (2015). Mechanisms of ATM Activation. Annu. Rev. Biochem..

[84] Bencokova Z., Kaufmann M.R., Pires I.M., Lecane P.S., Giaccia A.J., Hammond E.M. (2009). ATM Activation and Signaling under Hypoxic Conditions. Mol. Cell. Biol..

[85] Fallone F., Britton S., Nieto L., Salles B., Muller C. (2013). ATR Controls Cellular Adaptation to Hypoxia through Positive Regulation of Hypoxia-Inducible Factor 1 (HIF-1) Expression. Oncogene.

[86] Manic G., Obrist F., Sistigu A., Vitale I. (2015). Trial Watch: Targeting ATM–CHK2 and ATR–CHK1 Pathways for Anticancer Therapy. Mol. Cell. Oncol..

[87] Gibson S.L., Bindra R.S., Glazer P.M. (2005). Hypoxia-Induced Phosphorylation of Chk2 in an Ataxia Telangiectasia Mutated–Dependent Manner. Cancer Res..

[88] Basu S., Majumder S., Bhowal A., Ghosh A., Naskar S., Nandy S., Mukherjee S., Sinha R.K., Basu K., Karmakar D. (2015). A Study of Molecular Signals Deregulating Mismatch Repair Genes in Prostate Cancer Compared to Benign Prostatic Hyperplasia. PLoS ONE.

[89] Bindra R.S., Glazer P.M. (2007). Repression of RAD51 Gene Expression by E2F4/P130 Complexes in Hypoxia. Oncogene.

[90] Chan N., Koritzinsky M., Zhao H., Bindra R., Glazer P.M., Powell S., Belmaaza A., Wouters B., Bristow R.G. (2008). Chronic Hypoxia Decreases Synthesis of Homologous Recombination Proteins to Offset Chemoresistance and Radioresistance. Cancer Res..

[91] Valeri N., Gasparini P., Fabbri M., Braconi C., Veronese A., Lovat F., Adair B., Vannini I., Fanini F., Bottoni A. (2010). Modulation of Mismatch Repair and Genomic Stability by MiR-155. Proc. Natl. Acad. Sci. USA.

[92] Crosby M.E., Kulshreshtha R., Ivan M., Glazer P.M. (2009). MicroRNA Regulation of DNA Repair Gene Expression in Hypoxic Stress. Cancer Res..

[93] Lu Y., Chu A., Turker M.S., Glazer P.M. (2011). Hypoxia-Induced Epigenetic Regulation and Silencing of the *BRCA1* Promoter. Mol. Cell. Biol..

[94] Sulkowski P.L., Corso C.D., Robinson N.D., Scanlon S.E., Purshouse K.R., Bai H., Liu Y., Sundaram R.K., Hegan D.C., Fons N.R. (2017). 2-Hydroxyglutarate Produced by Neomorphic IDH Mutations Suppresses Homologous Recombination and Induces PARP Inhibitor Sensitivity. Sci. Transl. Med..

[95] Xiang K., Jendrossek V., Matschke J. (2020). Oncometabolites and the Response to Radiotherapy. Radiat. Oncol..

[96] Bouquet F., Ousset M., Biard D., Fallone F., Dauvillier S., Frit P., Salles B., Muller C. (2011). A DNA-Dependent Stress Response Involving DNA-PK Occurs in Hypoxic Cells and Contributes to Cellular Adaptation to Hypoxia. J. Cell Sci..

[97] Graham T.G.W., Walter J.C., Loparo J.J. (2016). Two-Stage Synapsis of DNA Ends during Non-Homologous End Joining. Mol. Cell.

[98] Bhandari V., Hoey C., Liu L.Y., Lalonde E., Ray J., Livingstone J., Lesurf R., Shiah Y.-J., Vujcic T., Huang X. (2019). Molecular Landmarks of Tumor Hypoxia across Cancer Types. Nat. Genet..

[99] Kaelin W.G., Ratcliffe P.J. (2008). Oxygen Sensing by Metazoans: The Central Role of the HIF Hydroxylase Pathway. Mol. Cell.

[100] Ehteshamipour S., Mohebbi S., Rahimi E., Shabani Sadr N.K., Mohamad Soltani B., Behmanesh M. (2024). HIF1α Contribution to the NHEJ DNA Repair Pathway Through Decreased Expression of XRCC4. Jentashapir J. Cell. Mol. Biol..

[101] Gomel R., Xiang C., Finniss S., Lee H.K., Lu W., Okhrimenko H., Brodie C. (2007). The Localization of Protein Kinase C δ in Different Subcellular Sites Affects Its Proapoptotic and Antiapoptotic Functions and the Activation of Distinct Downstream Signaling Pathways. Mol. Cancer Res..

[102] Luo Y., Li M., Zuo X., Basourakos S., Zhang J., Zhao J., Han Y., Lin Y., Wang Y., Jiang Y. (2018). Β-catenin Nuclear Translocation Induced by HIF-1α Overexpression Leads to the Radioresistance of Prostate Cancer. Int. J. Oncol..

[103] Zhang J., Zhang Q. (2018). VHL and Hypoxia Signaling: Beyond HIF in Cancer. Biomedicines.

[104] Artemov A.V., Zhigalova N., Zhenilo S., Mazur A.M., Prokhortchouk E.B. (2018). VHL Inactivation without Hypoxia Is Sufficient to Achieve Genome Hypermethylation. Sci. Rep..

[105] Rademakers S.E., Lok J., van der Kogel A.J., Bussink J., Kaanders J.H. (2011). Metabolic Markers in Relation to Hypoxia; Staining Patterns and Colocalization of Pimonidazole, HIF-1α, CAIX, LDH-5, GLUT-1, MCT1 and MCT4. BMC Cancer.

[106] Islam S., Mukherjee C. (2023). Molecular Regulation of Hypoxia through the Lenses of Noncoding RNAs and Epitranscriptome. Wiley Interdiscip. Rev. RNA.

[107] Sawai S., Wong P.-F., Ramasamy T.S. (2022). Hypoxia-Regulated MicroRNAs: The Molecular Drivers of Tumor Progression. Crit. Rev. Biochem. Mol. Biol..

[108] Godet I., Doctorman S., Wu F., Gilkes D.M. (2022). Detection of Hypoxia in Cancer Models: Significance, Challenges, and Advances. Cells.

[109] Zhao Z., Mu H., Li Y., Liu Y., Zou J., Zhu Y. (2020). Clinicopathological and Prognostic Value of Hypoxia-Inducible Factor-1α in Breast Cancer: A Meta-Analysis Including 5177 Patients. Clin. Transl. Oncol..

[110] Khojastehnezhad M.A., Seyedi S.M.R., Raoufi F., Asoodeh A. (2022). Association of Hypoxia-Inducible Factor 1 Expressions with Prognosis Role as a Survival Prognostic Biomarker in the Patients with Osteosarcoma: A Meta-Analysis. Expert Rev. Mol. Diagn..

[111] Li Z., Wei R., Yao S., Meng F., Kong L. (2024). HIF-1A as a Prognostic Biomarker Related to Invasion, Migration and Immunosuppression of Cervical Cancer. Heliyon.

[112] Qin J., Liu Y., Lu Y., Liu M., Li M., Li J., Wu L. (2017). Hypoxia-Inducible Factor 1 Alpha Promotes Cancer Stem Cells-like Properties in Human Ovarian Cancer Cells by Upregulating SIRT1 Expression. Sci. Rep..

[113] Chamie K., Klöpfer P., Bevan P., Störkel S., Said J., Fall B., Belldegrun A.S., Pantuck A.J. (2015). Carbonic Anhydrase-IX Score Is a Novel Biomarker That Predicts Recurrence and Survival for High-Risk, Nonmetastatic Renal Cell Carcinoma: Data from the Phase III ARISER Clinical Trial. Urol. Oncol. Semin. Orig. Investig..

[114] van Kuijk S.J.A., Yaromina A., Houben R., Niemans R., Lambin P., Dubois L.J. (2016). Prognostic Significance of Carbonic Anhydrase IX Expression in Cancer Patients: A Meta-Analysis. Front. Oncol..

[115] Tanaka K., Sugisaka J., Shiraishi Y., Watanabe Y., Daga H., Azuma K., Nishino K., Mori M., Ota T., Saito H. (2025). Serum VEGF-A as a Biomarker for the Addition of Bevacizumab to Chemo-Immunotherapy in Metastatic NSCLC. Nat. Commun..

[116] Han Y.J., Liu S., Hardeman A., Rajagopal P.S., Mueller J., Khramtsova G., Sanni A., Ajani M., Clayton W., Hurley I.W. (2024). The VEGF-Hypoxia Signature Is Upregulated in Basal-like Breast Tumors from Women of African Ancestry and Associated with Poor Outcomes in Breast Cancer. Clin. Cancer Res..

[117] Raleigh J.A., Calkins-Adams D.P., Rinker L.H., Ballenger C.A., Weissler M.C., Fowler W.C., Novotny D.B., Varia M.A. (1998). Hypoxia and Vascular Endothelial Growth Factor Expression in Human Squamous Cell Carcinomas Using Pimonidazole as a Hypoxia Marker. Cancer Res..

[118] Du J., Gu J., Deng J., Kong L., Guo Y., Jin C., Bao Y., Fu D., Li J. (2021). The Expression and Survival Significance of Glucose Transporter-1 in Pancreatic Cancer: Meta-Analysis, Bioinformatics Analysis and Retrospective Study. Cancer Investig..

[119] El-Benhawy S.A., Sakr O.A., Fahmy E.I., Ali R.A., Hussein M.S., Nassar E.M., Salem S.M., Abu-Samra N., Elzawawy S. (2022). Assessment of Serum Hypoxia Biomarkers Pre- and Post-Radiotherapy in Patients with Brain Tumors. J. Mol. Neurosci..

[120] Wang Y., Li Y., Jiang L., Ren X., Cheng B., Xia J. (2021). Prognostic Value of Glycolysis Markers in Head and Neck Squamous Cell Carcinoma: A Meta-Analysis. Aging.

[121] Serganova I., Rizwan A., Ni X., Thakur S.B., Vider J., Russell J., Blasberg R., Koutcher J.A. (2011). Metabolic Imaging: A Link between Lactate Dehydrogenase A, Lactate, and Tumor Phenotype. Clin. Cancer Res..

[122] Radenkovic S., Milosevic Z., Konjevic G., Karadzic K., Rovcanin B., Buta M., Gopcevic K., Jurisic V. (2013). Lactate Dehydrogenase, Catalase, and Superoxide Dismutase in Tumor Tissue of Breast Cancer Patients in Respect to Mammographic Findings. Cell Biochem. Biophys.

[123] Han Y.-L., Chen L., Qin R., Wang G.-Q., Lin X.-H., Dai G.-H. (2019). Lysyl Oxidase and Hypoxia-Inducible Factor 1α: Biomarkers of Gastric Cancer. World J. Gastroenterol..

[124] Schietke R., Warnecke C., Wacker I., Schödel J., Mole D.R., Campean V., Amann K., Goppelt-Struebe M., Behrens J., Eckardt K.-U. (2010). The Lysyl Oxidases LOX and LOXL2 Are Necessary and Sufficient to Repress E-Cadherin in Hypoxia. J. Biol. Chem..

[125] Liu E., Li W., Jian L., Yin S., Yang S., Zhao H., Huang W., Zhang Y., Zhou H. (2023). Identification of LOX as a Candidate Prognostic Biomarker in Glioblastoma Multiforme. Transl. Oncol..

[126] Tang Y., Zhou X., Ji J., Chen L., Cao J., Luo J., Zhang S. (2015). High Expression Levels of MiR-21 and MiR-210 Predict Unfavorable Survival in Breast Cancer: A Systemic Review and Meta-Analysis. Int. J. Biol. Markers.

[127] Hu X., Peng Q., Zhu J., Shen Y., Lin K., Shen Y., Zhu Y. (2020). Identification of MiR-210 and Combination Biomarkers as Useful Agents in Early Screening Non-Small Cell Lung Cancer. Gene.

[128] Irlam-Jones J.J., Eustace A., Denley H., Choudhury A., Harris A.L., Hoskin P.J., West C.M.L. (2016). Expression of MiR-210 in Relation to Other Measures of Hypoxia and Prediction of Benefit from Hypoxia Modification in Patients with Bladder Cancer. Br. J. Cancer.

[129] Li M., Ma X., Li M., Zhang B., Huang J., Liu L., Wei Y. (2014). Prognostic Role of MicroRNA-210 in Various Carcinomas: A Systematic Review and Meta-Analysis. Dis. Markers.

[130] Ci X., Chen S., Zhu R., Zarif M., Jain R., Guo W., Ramotar M., Gong L., Xu W., Singh O. (2024). Oral Pimonidazole Unveils Clinicopathologic and Epigenetic Features of Hypoxic Tumour Aggressiveness in Localized Prostate Cancer. BMC Cancer.

[131] Ragnum H.B., Vlatkovic L., Lie A.K., Axcrona K., Julin C.H., Frikstad K.M., Hole K.H., Seierstad T., Lyng H. (2015). The Tumour Hypoxia Marker Pimonidazole Reflects a Transcriptional Programme Associated with Aggressive Prostate Cancer. Br. J. Cancer.

[132] Swartz J.E., Smits H.J.G., Philippens M.E.P., de Bree R., Kaanders J.H.A.M., Willems S.M. (2022). Correlation and Colocalization of HIF-1α and Pimonidazole Staining for Hypoxia in Laryngeal Squamous Cell Carcinomas: A Digital, Single-Cell-Based Analysis. Oral Oncol..

[133] Beckers C., Pruschy M., Vetrugno I. (2024). Tumor Hypoxia and Radiotherapy: A Major Driver of Resistance Even for Novel Radiotherapy Modalities. Semin. Cancer Biol..

[134] Minassian L.M., Cotechini T., Huitema E., Graham C.H. (2019). Hypoxia-Induced Resistance to Chemotherapy in Cancer. Adv. Exp. Med. Biol..

[135] Sørensen B.S., Horsman M.R. (2020). Tumor Hypoxia: Impact on Radiation Therapy and Molecular Pathways. Front. Oncol..

[136] Bouleftour W., Rowinski E., Louati S., Sotton S., Wozny A.-S., Moreno-Acosta P., Mery B., Rodriguez-Lafrasse C., Magne N. (2021). A Review of the Role of Hypoxia in Radioresistance in Cancer Therapy. Med. Sci. Monit..

[137] Marcu L.G., Toma Dasu I., Dasu A., Marcu L.G. (2015). The Six Rs of Head and Neck Cancer Radiotherapy. Contemporary Issues in Head and Neck Cancer Management.

[138] Meijer T.W.H., Kaanders J.H.A.M., Span P.N., Bussink J. (2012). Targeting Hypoxia, HIF-1, and Tumor Glucose Metabolism to Improve Radiotherapy Efficacy. Clin. Cancer Res..

[139] Rakotomalala A., Escande A., Furlan A., Meignan S., Lartigau E. (2021). Hypoxia in Solid Tumors: How Low Oxygenation Impacts the “Six Rs” of Radiotherapy. Front. Endocrinol.

[140] Ma Y., Hendershot L.M. (2004). The Role of the Unfolded Protein Response in Tumour Development: Friend or Foe?. Nat. Rev. Cancer.

[141] Tian Y., Lei Y., Wang Y., Lai J., Wang J., Xia F. (2023). Mechanism of Multidrug Resistance to Chemotherapy Mediated by P-glycoprotein (Review). Int. J. Oncol..

[142] Ozcan G. (2023). The Hypoxia-Inducible Factor-1α in Stemness and Resistance to Chemotherapy in Gastric Cancer: Future Directions for Therapeutic Targeting. Front. Cell Dev. Biol..

[143] Xia Y., Jiang L., Zhong T. (2018). The Role of HIF-1 & alpha; in Chemo-/Radioresistant Tumors. Onco Targets Ther..

[144] Zhao Q., Tan B.-B., Li Y., Fan L.-Q., Yang P.-G., Tian Y. (2016). Enhancement of Drug Sensitivity by Knockdown of HIF-1α in Gastric Carcinoma Cells. Oncol. Res. Featur. Preclin. Clin. Cancer Ther..

[145] Rohwer N., Dame C., Haugstetter A., Wiedenmann B., Detjen K., Schmitt C.A., Cramer T. (2010). Hypoxia-Inducible Factor 1α Determines Gastric Cancer Chemosensitivity via Modulation of P53 and NF-ΚB. PLoS ONE.

[146] Li X., Zhou Y., Li Y., Yang L., Ma Y., Peng X., Yang S., Liu J., Li H. (2019). Autophagy: A Novel Mechanism of Chemoresistance in Cancers. Biomed. Pharmacother..

[147] Liu C., Jin Y., Fan Z. (2021). The Mechanism of Warburg Effect-Induced Chemoresistance in Cancer. Front. Oncol..

[148] Sowa T., Menju T., Chen-Yoshikawa T.F., Takahashi K., Nishikawa S., Nakanishi T., Shikuma K., Motoyama H., Hijiya K., Aoyama A. (2017). Hypoxia-inducible Factor 1 Promotes Chemoresistance of Lung Cancer by Inducing Carbonic Anhydrase IX Expression. Cancer Med..

[149] Sebestyén A., Kopper L., Dankó T., Tímár J. (2021). Hypoxia Signaling in Cancer: From Basics to Clinical Practice. Pathol. Oncol. Res..

[150] Hielscher A., Gerecht S. (2015). Hypoxia and Free Radicals: Role in Tumor Progression and the Use of Engineering-Based Platforms to Address These Relationships. Free. Radic. Biol. Med..

[151] Doktorova H., Hrabeta J., Khalil M.A., Eckschlager T. (2015). Hypoxia-Induced Chemoresistance in Cancer Cells: The Role of Not Only HIF-1. Biomed. Pap..

[152] Burger R.M., Peisach J., Horwitz S.B. (1981). Activated Bleomycin. A Transient Complex of Drug, Iron, and Oxygen That Degrades DNA. J. Biol. Chem..

[153] Rouschop K.M., Dubois L.J., Keulers T.G., van den Beucken T., Lambin P., Bussink J., van der Kogel A.J., Koritzinsky M., Wouters B.G. (2013). PERK/EIF2α Signaling Protects Therapy Resistant Hypoxic Cells through Induction of Glutathione Synthesis and Protection against ROS. Proc. Natl. Acad. Sci. USA.

[154] Salaroglio I.C., Panada E., Moiso E., Buondonno I., Provero P., Rubinstein M., Kopecka J., Riganti C. (2017). PERK Induces Resistance to Cell Death Elicited by Endoplasmic Reticulum Stress and Chemotherapy. Mol. Cancer.

[155] Bi M., Naczki C., Koritzinsky M., Fels D., Blais J., Hu N., Harding H., Novoa I., Varia M., Raleigh J. (2005). ER Stress-Regulated Translation Increases Tolerance to Extreme Hypoxia and Promotes Tumor Growth. EMBO J..

[156] Romero-Ramirez L., Cao H., Nelson D., Hammond E., Lee A.-H., Yoshida H., Mori K., Glimcher L.H., Denko N.C., Giaccia A.J. (2004). XBP1 Is Essential for Survival under Hypoxic Conditions and Is Required for Tumor Growth. Cancer Res..

[157] Augustin R.C., Delgoffe G.M., Najjar Y.G. (2020). Characteristics of the Tumor Microenvironment That Influence Immune Cell Functions: Hypoxia, Oxidative Stress, Metabolic Alterations. Cancers.

[158] Terry S., Buart S., Chouaib S. (2017). Hypoxic Stress-Induced Tumor and Immune Plasticity, Suppression, and Impact on Tumor Heterogeneity. Front. Immunol..

[159] Phan A.T., Goldrath A.W. (2015). Hypoxia-Inducible Factors Regulate T Cell Metabolism and Function. Mol. Immunol..

[160] Tao J.-H., Barbi J., Pan F. (2015). Hypoxia-Inducible Factors in T Lymphocyte Differentiation and Function. A Review in the Theme: Cellular Responses to Hypoxia. Am. J. Physiol. Cell Physiol..

[161] Wang Y., Wang H., Yao H., Li C., Fang J.-Y., Xu J. (2018). Regulation of PD-L1: Emerging Routes for Targeting Tumor Immune Evasion. Front. Pharmacol..

[162] Robainas M., Otano R., Bueno S., Ait-Oudhia S. (2017). Understanding the Role of PD-L1/PD1 Pathway Blockade and Autophagy in Cancer Therapy. Onco Targets Ther..

[163] Steingold J.M., Hatfield S.M. (2020). Targeting Hypoxia-A2A Adenosinergic Immunosuppression of Antitumor T Cells During Cancer Immunotherapy. Front. Immunol..

[164] Graham K., Unger E. (2018). Overcoming Tumor Hypoxia as a Barrier to Radiotherapy, Chemotherapy and Immunotherapy in Cancer Treatment. Int. J. Nanomed..

[165] (2020). Editor’s Pick: Tumour-Associated Hypoxia: Can We Give Chimeric Antigen Receptor T Cells More Breathing Space?. Eur. Med. J..

[166] Schurich A., Magalhaes I., Mattsson J. (2019). Metabolic Regulation of CAR T Cell Function by the Hypoxic Microenvironment in Solid Tumors. Immunotherapy.

[167] Huang Y., Kim B.Y.S., Chan C.K., Hahn S.M., Weissman I.L., Jiang W. (2018). Improving Immune–Vascular Crosstalk for Cancer Immunotherapy. Nat. Rev. Immunol..

[168] Park J.A., Espinosa-Cotton M., Guo H., Monette S., Cheung N.-K.V. (2023). Targeting Tumor Vasculature to Improve Antitumor Activity of T Cells Armed Ex Vivo with T Cell Engaging Bispecific Antibody. J. Immunother. Cancer.

[169] Hendry S.A., Farnsworth R.H., Solomon B., Achen M.G., Stacker S.A., Fox S.B. (2016). The Role of the Tumor Vasculature in the Host Immune Response: Implications for Therapeutic Strategies Targeting the Tumor Microenvironment. Front. Immunol..

[170] Li Y., Zhao L., Li X.-F. (2021). Targeting Hypoxia: Hypoxia-Activated Prodrugs in Cancer Therapy. Front. Oncol..

[171] Baran N., Konopleva M. (2017). Molecular Pathways: Hypoxia-Activated Prodrugs in Cancer Therapy. Clin. Cancer Res..

[172] Li Y., Zhao L., Li X.-F. (2021). The Hypoxia-Activated Prodrug TH-302: Exploiting Hypoxia in Cancer Therapy. Front. Pharmacol..

[173] van der Wiel A.M.A., Jackson-Patel V., Niemans R., Yaromina A., Liu E., Marcus D., Mowday A.M., Lieuwes N.G., Biemans R., Lin X. (2021). Selectively Targeting Tumor Hypoxia with the Hypoxia-Activated Prodrug CP-506. Mol. Cancer Ther..

[174] Musleh Ud Din S., Streit S.G., Huynh B.T., Hana C., Abraham A.-N., Hussein A. (2024). Therapeutic Targeting of Hypoxia-Inducible Factors in Cancer. Int. J. Mol. Sci..

[175] Abi-Jaoudeh N., Dayyani F., Chen P.J., Fernando D., Fidelman N., Javan H., Liang P.-C., Hwang J.-I., Imagawa D.K. (2021). Phase I Trial on Arterial Embolization with Hypoxia Activated Tirapazamine for Unresectable Hepatocellular Carcinoma. J. Hepatocell. Carcinoma.

[176] Liu C.-H., Peng C.-M., Hwang J.-I., Liang P.-C., Chen P.-J., Abi-Jaoudeh N., Giiang L.-H., Tyan Y.-S. (2022). Phase I Dose-Escalation Study of Tirapazamine Chemoembolization for Unresectable Early- and Intermediate-Stage Hepatocellular Carcinoma. J. Vasc. Interv. Radiol..

[177] Huynh K.N., Rao S., Roth B., Bryan T., Fernando D.M., Dayyani F., Imagawa D., Abi-Jaoudeh N. (2023). Targeting Hypoxia-Inducible Factor-1α for the Management of Hepatocellular Carcinoma. Cancers.

[178] Benito J., Shi Y., Szymanska B., Carol H., Boehm I., Lu H., Konoplev S., Fang W., Zweidler-McKay P.A., Campana D. (2011). Pronounced Hypoxia in Models of Murine and Human Leukemia: High Efficacy of Hypoxia-Activated Prodrug PR-104. PLoS ONE.

[179] Konopleva M., Thall P.F., Yi C.A., Borthakur G., Coveler A., Bueso-Ramos C., Benito J., Konoplev S., Gu Y., Ravandi F. (2015). Phase I/II Study of the Hypoxia-Activated Prodrug PR104 in Refractory/Relapsed Acute Myeloid Leukemia and Acute Lymphoblastic Leukemia. Haematologica.

[180] Singleton R.S., Guise C.P., Ferry D.M., Pullen S.M., Dorie M.J., Brown J.M., Patterson A.V., Wilson W.R. (2009). DNA Cross-Links in Human Tumor Cells Exposed to the Prodrug PR-104A: Relationships to Hypoxia, Bioreductive Metabolism, and Cytotoxicity. Cancer Res..

[181] Yaromina A., Koi L., Schuitmaker L., van der Wiel A.M.-M.A., Dubois L.J., Krause M., Lambin P. (2023). Overcoming Radioresistance with the Hypoxia-Activated Prodrug CP-506: A Pre-Clinical Study of Local Tumour Control Probability. Radiother. Oncol..

[182] Brenner A., Zuniga R., Sun J.D., Floyd J., Hart C.P., Kroll S., Fichtel L., Cavazos D., Caflisch L., Gruslova A. (2018). Hypoxia-Activated Evofosfamide for Treatment of Recurrent Bevacizumab-Refractory Glioblastoma: A Phase I Surgical Study. Neuro. Oncol..

[183] Brenner A.J., Floyd J., Fichtel L., Michalek J., Kanakia K.P., Huang S., Reardon D., Wen P.Y., Lee E.Q. (2021). Phase 2 Trial of Hypoxia Activated Evofosfamide (TH302) for Treatment of Recurrent Bevacizumab-Refractory Glioblastoma. Sci. Rep..

[184] Sun J.D., Liu Q., Ahluwalia D., Ferraro D.J., Wang Y., Jung D., Matteucci M.D., Hart C.P. (2016). Comparison of Hypoxia-Activated Prodrug Evofosfamide (TH-302) and Ifosfamide in Preclinical Non-Small Cell Lung Cancer Models. Cancer Biol. Ther..

[185] Laubach J.P., Liu C.-J., Raje N.S., Yee A.J., Armand P., Schlossman R.L., Rosenblatt J., Hedlund J., Martin M., Reynolds C. (2019). Phase I/II Study of Evofosfamide, A Hypoxia-Activated Prodrug with or without Bortezomib in Subjects with Relapsed/Refractory Multiple Myeloma. Clin. Cancer Res..

[186] Sun J.D., Liu Q., Ahluwalia D., Li W., Meng F., Wang Y., Bhupathi D., Ruprell A.S., Hart C.P. (2015). Efficacy and Safety of the Hypoxia-Activated Prodrug TH-302 in Combination with Gemcitabine and Nab-Paclitaxel in Human Tumor Xenograft Models of Pancreatic Cancer. Cancer Biol. Ther..

[187] Badar T., Handisides D.R., Benito J.M., Richie M.A., Borthakur G., Jabbour E., Harutyunyan K., Konoplev S., Faderl S., Kroll S. (2016). Phase I Study of Evofosfamide, an Investigational Hypoxia-activated Prodrug, in Patients with Advanced Leukemia. Am. J. Hematol..

[188] McLean L.S., Morris T.A., Gramza A., Liu S., Khan S.A., Colevas A.D., Pearce T., Rischin D. (2022). A Phase II Study of Tarloxotinib (a Hypoxia Activated Prodrug of a Pan-Erb Tyrosine Kinase Inhibitor) in Patients with Recurrent or Metastatic Squamous Cell Carcinoma of the Head and Neck or Skin. Investig. New Drugs.

[189] Estrada-Bernal A., Le A.T., Doak A.E., Tirunagaru V.G., Silva S., Bull M.R., Smaill J.B., Patterson A.V., Kim C., Liu S.V. (2021). Tarloxotinib Is a Hypoxia-Activated Pan-HER Kinase Inhibitor Active Against a Broad Range of HER-Family Oncogenes. Clin. Cancer Res..

[190] Xu P., Huang J.-M., Ren Y., Zha X., Deng B.-F., Wu J.-H., Lang J.-Y. (2010). Regulation of Hypoxia-Induced MRNA Expressions of HIF-1alpha?And Osteopontin and in Vitro Radiosensitization by Tirapazamine in Human Nasopharyngeal Carcinoma HNE-1 and CNE-1 Cells. Chin. J. Cancer.

[191] Meng F., Evans J.W., Bhupathi D., Banica M., Lan L., Lorente G., Duan J.-X., Cai X., Mowday A.M., Guise C.P. (2012). Molecular and Cellular Pharmacology of the Hypoxia-Activated Prodrug TH-302. Mol. Cancer Ther..

[192] Spiegelberg L., Houben R., Niemans R., de Ruysscher D., Yaromina A., Theys J., Guise C.P., Smaill J.B., Patterson A.V., Lambin P. (2019). Hypoxia-Activated Prodrugs and (Lack of) Clinical Progress: The Need for Hypoxia-Based Biomarker Patient Selection in Phase III Clinical Trials. Clin. Transl. Radiat. Oncol..

[193] Jayaprakash P., Ai M., Liu A., Budhani P., Bartkowiak T., Sheng J., Ager C., Nicholas C., Jaiswal A.R., Sun Y. (2018). Targeted Hypoxia Reduction Restores T Cell Infiltration and Sensitizes Prostate Cancer to Immunotherapy. J. Clin. Investig..

[194] Jamieson S.M.F., Tsai P., Kondratyev M.K., Budhani P., Liu A., Senzer N.N., Chiorean E.G., Jalal S.I., Nemunaitis J.J., Kee D. (2018). Evofosfamide for the Treatment of Human Papillomavirus-Negative Head and Neck Squamous Cell Carcinoma. JCI Insight.

[195] Hegde A., Jayaprakash P., Couillault C.A., Piha-Paul S., Karp D., Rodon J., Pant S., Fu S., Dumbrava E.E., Yap T.A. (2021). A Phase I Dose-Escalation Study to Evaluate the Safety and Tolerability of Evofosfamide in Combination with Ipilimumab in Advanced Solid Malignancies. Clin. Cancer Res..

[196] Palmer B.D., Wilson W.R., Atwell G.J., Schultz D., Xu X.Z., Denny W.A. (1994). Hypoxia-Selective Antitumor Agents. 9. Structure-Activity Relationships for Hypoxia-Selective Cytotoxicity among Analogs of 5-[N,N-Bis(2-Chloroethyl)Amino]-2,4-Dinitrobenzamide. J. Med. Chem..

[197] Liu S.V., Villaruz L.C., Lee V.H.F., Zhu V.W., Baik C.S., Sacher A., McCoach C.E., Nguyen D., Li J.Y.-C., Pacheco J.M. (2020). LBA61 First Analysis of RAIN-701: Study of Tarloxotinib in Patients with Non-Small Cell Lung Cancer (NSCLC) EGFR Exon 20 Insertion, HER2-Activating Mutations & Other Solid Tumours with NRG1/ERBB Gene Fusions. Ann. Oncol..

[198] Ciepła J., Smolarczyk R. (2024). Tumor Hypoxia Unveiled: Insights into Microenvironment, Detection Tools and Emerging Therapies. Clin. Exp. Med..

[199] Moen I., Stuhr L.E.B. (2012). Hyperbaric Oxygen Therapy and Cancer—A Review. Target. Oncol..

[200] Ortega M.A., Fraile-Martinez O., García-Montero C., Callejón-Peláez E., Sáez M.A., Álvarez-Mon M.A., García-Honduvilla N., Monserrat J., Álvarez-Mon M., Bujan J. (2021). A General Overview on the Hyperbaric Oxygen Therapy: Applications, Mechanisms and Translational Opportunities. Medicina.

[201] Stirnemann J., Serratrice J., Mann T., Louge P., Christophe C., Samii K., Pignel R., Agoritsas T., Ansari M., Cannas G. (2024). Protocol for a Multicentric, Double-Blind, Randomised Controlled Trial of Hyperbaric Oxygen Therapy (HBOT) versus Sham for Treating Vaso-Occlusive Crisis (VOC) in Sickle Cell Disease (SCD) in Patients Aged 8 Years or Older (HBOT-SCD Study). BMJ Open.

[202] Stępień K., Ostrowski R.P., Matyja E. (2016). Hyperbaric Oxygen as an Adjunctive Therapy in Treatment of Malignancies, Including Brain Tumours. Med. Oncol..

[203] Canarslan Demir K., Avci A.U., Ozgok Kangal M.K., Ceylan B., Abayli S.Y., Ozler I., Yilmaz K.B. (2025). Hyperbaric Oxygen Therapy for Managing Cancer Treatment Complications: A Safety Evaluation. Medicina.

[204] Granowitz E.V., Tonomura N., Benson R.M., Katz D.M., Band V., Makari-Judson G.P., Osborne B.A. (2005). Hyperbaric Oxygen Inhibits Benign and Malignant Human Mammary Epithelial Cell Proliferation. Anticancer Res..

[205] Machado V., da Rocha J.R., Parra R., Feitosa M., Leite C., Minto S., Garcia S., Cunha T., Feres O. (2023). Hyperbaric Oxygen Therapy Increases the Effect of 5-Fluorouracil Chemotherapy on Experimental Colorectal Cancer in Mice. Med. Gas. Res..

[206] Chen S.-Y., Tsuneyama K., Yen M.-H., Lee J.-T., Chen J.-L., Huang S.-M. (2021). Hyperbaric Oxygen Suppressed Tumor Progression through the Improvement of Tumor Hypoxia and Induction of Tumor Apoptosis in A549-Cell-Transferred Lung Cancer. Sci. Rep..

[207] Fernández E., Morillo V., Salvador M., Santafé A., Beato I., Rodríguez M., Ferrer C. (2021). Hyperbaric Oxygen and Radiation Therapy: A Review. Clin. Transl. Oncol..

[208] Yuen C.-M., Tsai H.-P., Tseng T.-T., Tseng Y.-L., Lieu A.-S., Kwan A.-L., Chang A.Y.W. (2023). Hyperbaric Oxygen Therapy Adjuvant Chemotherapy and Radiotherapy through Inhibiting Stemness in Glioblastoma. Curr. Issues Mol. Biol..

[209] Poff A.M., Ward N., Seyfried T.N., Arnold P., D’Agostino D.P. (2015). Non-Toxic Metabolic Management of Metastatic Cancer in VM Mice: Novel Combination of Ketogenic Diet, Ketone Supplementation, and Hyperbaric Oxygen Therapy. PLoS ONE.

[210] Chen Y.-C., Chen S.-Y., Ho P.-S., Lin C.-H., Cheng Y.-Y., Wang J.-K., Sytwu H.-K. (2007). Apoptosis of T-Leukemia and B-Myeloma Cancer Cells Induced by Hyperbaric Oxygen Increased Phosphorylation of P38 MAPK. Leuk. Res..

[211] Liu X., Ye N., Xiao C., Wang X., Li S., Deng Y., Yang X., Li Z., Yang X. (2021). Hyperbaric Oxygen Regulates Tumor Microenvironment and Boosts Commercialized Nanomedicine Delivery for Potent Eradication of Cancer Stem-like Cells. Nano Today.

[212] Ren Z., Wang R., Wang C., Ren X., Li D., Liu Y., Yu Q. (2024). Key Genes Involved in the Beneficial Mechanism of Hyperbaric Oxygen for Glioblastoma and Predictive Indicators of Hyperbaric Oxygen Prolonging Survival in Glioblastoma Patients. Curr. Med. Sci..

[213] Jansman M.M.T., Hosta-Rigau L. (2018). Recent and Prominent Examples of Nano- and Microarchitectures as Hemoglobin-Based Oxygen Carriers. Adv. Colloid Interface Sci..

[214] Kawasoe Y., Yokouchi M., Ueno Y., Iwaya H., Yoshida H., Komiya S. (2009). Hyperbaric Oxygen as a Chemotherapy Adjuvant in the Treatment of Osteosarcoma. Oncol. Rep..

[215] Wang P., Wang X.-Y., Man C.-F., Gong D.-D., Fan Y. (2023). Advances in Hyperbaric Oxygen to Promote Immunotherapy through Modulation of the Tumor Microenvironment. Front. Oncol..

[216] Moen I., Øyan A.M., Kalland K.-H., Tronstad K.J., Akslen L.A., Chekenya M., Sakariassen P.Ø., Reed R.K., Stuhr L.E.B. (2009). Hyperoxic Treatment Induces Mesenchymal-to-Epithelial Transition in a Rat Adenocarcinoma Model. PLoS ONE.

[217] Alagoz T., Buller R.E., Anderson B., Terrell K.L., Squatrito R.C., Niemann T.H., Tatman D.J., Jebson P. (1995). Evaluation of Hyperbaric Oxygen as a Chemosensitizer in the Treatment of Epithelial Ovarian Cancer in Xenografts in Mice. Cancer.

[218] Al-Waili N.S., Butler G.J., Beale J., Hamilton R.W., Lee B.Y., Lucas P. (2005). Hyperbaric Oxygen and Malignancies: A Potential Role in Radiotherapy, Chemotherapy, Tumor Surgery and Phototherapy. Med. Sci. Monit..

[219] Bui B.P., Nguyen P.L., Lee K., Cho J. (2022). Hypoxia-Inducible Factor-1: A Novel Therapeutic Target for the Management of Cancer, Drug Resistance, and Cancer-Related Pain. Cancers.

[220] Infantino V., Santarsiero A., Convertini P., Todisco S., Iacobazzi V. (2021). Cancer Cell Metabolism in Hypoxia: Role of HIF-1 as Key Regulator and Therapeutic Target. Int. J. Mol. Sci..

[221] Norris R.E., Shusterman S., Gore L., Muscal J.A., Macy M.E., Fox E., Berkowitz N., Buchbinder A., Bagatell R. (2014). Phase 1 Evaluation of EZN-2208, a Polyethylene Glycol Conjugate of SN38, in Children Adolescents and Young Adults with Relapsed or Refractory Solid Tumors. Pediatr. Blood Cancer.

[222] Jeong W., Park S.R., Rapisarda A., Fer N., Kinders R.J., Chen A., Melillo G., Turkbey B., Steinberg S.M., Choyke P. (2014). Weekly EZN-2208 (PEGylated SN-38) in Combination with Bevacizumab in Patients with Refractory Solid Tumors. Invest New Drugs.

[223] Kummar S., Raffeld M., Juwara L., Horneffer Y., Strassberger A., Allen D., Steinberg S.M., Rapisarda A., Spencer S.D., Figg W.D. (2011). Multihistology, Target-Driven Pilot Trial of Oral Topotecan as an Inhibitor of Hypoxia-Inducible Factor-1α in Advanced Solid Tumors. Clin. Cancer Res..

[224] Jeong W., Rapisarda A., Park S.R., Kinders R.J., Chen A., Melillo G., Turkbey B., Steinberg S.M., Choyke P., Doroshow J.H. (2014). Pilot Trial of EZN-2968, an Antisense Oligonucleotide Inhibitor of Hypoxia-Inducible Factor-1 Alpha (HIF-1α), in Patients with Refractory Solid Tumors. Cancer Chemother. Pharmacol..

[225] Ruan J., Zain J., Palmer B., Jovanovic B., Mi X., Swaroop A., Winter J.N., Gordon L.I., Karmali R., Moreira J. (2023). Multicenter Phase 2 Study of Romidepsin plus Lenalidomide for Previously Untreated Peripheral T-Cell Lymphoma. Blood Adv..

[226] Iyer S.P., Huen A., Ai W.Z., Jagadeesh D., Lechowicz M.J., Okada C., Feldman T.A., Ghione P., Alderuccio J.P., Champion R. (2023). Safety and Efficacy of Tenalisib in Combination with Romidepsin in Patients with Relapsed/Refractory T-Cell Lymphoma: Results from a Phase I/II Open-Label Multicenter Study. Haematologica.

[227] Foley N., Riedell P.A., Bartlett N.L., Cashen A.F., Kahl B.S., Fehniger T.A., Fischer A., Moreno C., Liu J., Carson K.R. (2025). A Phase I Study of Romidepsin in Combination with Gemcitabine, Oxaliplatin, and Dexamethasone in Patients with Relapsed or Refractory Aggressive Lymphomas Enriched for T-Cell Lymphomas. Clin. Lymphoma Myeloma Leuk..

[228] Horwitz S.M., Nirmal A.J., Rahman J., Xu R., Drill E., Galasso N., Ganesan N., Davey T., Hancock H., Perez L. (2024). Duvelisib plus Romidepsin in Relapsed/Refractory T Cell Lymphomas: A Phase 1b/2a Trial. Nat. Med..

[229] Pili R., Quinn D.I., Adra N., Logan T., Colligan S., Burney H.N., Hahn N.M. (2025). A Phase I, Open Label, Dose Finding Study to Evaluate Safety, Pharmacodynamics and Efficacy of Pembrolizumab in Combination with Vorinostat in Patients with Advanced Prostate, Renal or Urothelial Carcinoma. Cancer Med..

[230] Godfrey J., Mei M., Chen L., Song J.Y., Bedell V., Budde E., Armenian S., Puverel S., Nikolaenko L., Chen R. (2023). Results from a Phase I Trial of Pembrolizumab plus Vorinostat in Relapsed/Refractory B-Cell Non-Hodgkin Lymphoma. Haematologica.

[231] Stitzlein L.M., Baig M.U., Chandra J., McGovern S., Paulino A., Ketonen L.M., Khatua S., Zaky W. (2025). Phase I Study of Vorinostat and Temsirolimus in Newly Diagnosed or Progressive Diffuse Intrinsic Pontine Glioma. Pediatr. Blood Cancer.

[232] Arora S.P., Tenner L., Sarantopoulos J., Morris J., Liu Q., Mendez J.A., Curiel T., Michalek J., Mahalingam D. (2022). Modulation of Autophagy: A Phase II Study of Vorinostat plus Hydroxychloroquine versus Regorafenib in Chemotherapy-Refractory Metastatic Colorectal Cancer (MCRC). Br. J. Cancer.

[233] Krug L.M., Kindler H.L., Calvert H., Manegold C., Tsao A.S., Fennell D., Öhman R., Plummer R., Eberhardt W.E.E., Fukuoka K. (2015). Vorinostat in Patients with Advanced Malignant Pleural Mesothelioma Who Have Progressed on Previous Chemotherapy (VANTAGE-014): A Phase 3, Double-Blind, Randomised, Placebo-Controlled Trial. Lancet Oncol..

[234] Jenner M.W., Pawlyn C., Davies F.E., Menzies T., Hockaday A., Olivier C., Jones J.R., Karunanithi K., Lindsay J., Kishore B. (2022). The Addition of Vorinostat to Lenalidomide Maintenance for Patients with Newly Diagnosed Multiple Myeloma of All Ages: Results from ‘Myeloma XI’, a Multicentre, Open-label, Randomised, Phase III Trial. Br. J. Haematol..

[235] Garcia-Manero G., Podoltsev N.A., Othus M., Pagel J.M., Radich J.P., Fang M., Rizzieri D.A., Marcucci G., Strickland S.A., Litzow M.R. (2024). A Randomized Phase III Study of Standard versus High-Dose Cytarabine with or without Vorinostat for AML. Leukemia.

[236] Laubach J.P., Schjesvold F., Mariz M., Dimopoulos M.A., Lech-Maranda E., Spicka I., Hungria V.T.M., Shelekhova T., Abdo A., Jacobasch L. (2021). Efficacy and Safety of Oral Panobinostat plus Subcutaneous Bortezomib and Oral Dexamethasone in Patients with Relapsed or Relapsed and Refractory Multiple Myeloma (PANORAMA 3): An Open-Label, Randomised, Phase 2 Study. Lancet Oncol..

[237] San-Miguel J.F., Richardson P.G., Günther A., Sezer O., Siegel D., Bladé J., LeBlanc R., Sutherland H., Sopala M., Mishra K.K. (2013). Phase Ib study of panobinostat and bortezomib in relapsed or relapsed and refractory multiple myeloma. J. Clin. Oncol..

[238] Monje M., Cooney T., Glod J., Huang J., Peer C.J., Faury D., Baxter P., Kramer K., Lenzen A., Robison N.J. (2023). Phase I Trial of Panobinostat in Children with Diffuse Intrinsic Pontine Glioma: A Report from the Pediatric Brain Tumor Consortium (PBTC-047). Neuro Oncol..

[239] San-Miguel J.F., Hungria V.T.M., Yoon S.-S., Beksac M., Dimopoulos M.A., Elghandour A., Jedrzejczak W.W., Günther A., Nakorn T.N., Siritanaratkul N. (2014). Panobinostat plus Bortezomib and Dexamethasone versus Placebo plus Bortezomib and Dexamethasone in Patients with Relapsed or Relapsed and Refractory Multiple Myeloma: A Multicentre, Randomised, Double-Blind Phase 3 Trial. Lancet Oncol..

[240] Richardson P.G., Schlossman R.L., Roy A.N., Panneerselvam A., Acharyya S., Sopala M., Lonial S. (2018). Patient-reported Outcomes of Multiple Myeloma Patients Treated with Panobinostat after ≥2 Lines of Therapy Based on the International Phase 3, Randomized, Double-blind, Placebo-controlled PANORAMA-1 Trial. Br. J. Haematol..

[241] Heath E.I., Hillman D.W., Vaishampayan U., Sheng S., Sarkar F., Harper F., Gaskins M., Pitot H.C., Tan W., Ivy S.P. (2008). Carducci, M.A.; Erlichman, C.; Liu, G. A Phase II Trial of 17-Allylamino-17-Demethoxygeldanamycin in Patients with Hormone-Refractory Metastatic Prostate Cancer. Clin. Cancer Res..

[242] Wahner Hendrickson A.E., Oberg A.L., Glaser G., Camoriano J.K., Peethambaram P.P., Colon-Otero G., Erlichman C., Ivy S.P., Kaufmann S.H., Karnitz L.M. (2012). A Phase II Study of Gemcitabine in Combination with Tanespimycin in Advanced Epithelial Ovarian and Primary Peritoneal Carcinoma. Gynecol. Oncol..

[243] Iyer G., Morris M.J., Rathkopf D., Slovin S.F., Steers M., Larson S.M., Schwartz L.H., Curley T., DeLaCruz A., Ye Q. (2012). A Phase I Trial of Docetaxel and Pulse-Dose 17-Allylamino-17-Demethoxygeldanamycin in Adult Patients with Solid Tumors. Cancer Chemother. Pharmacol..

[244] Walker A.R., Klisovic R., Johnston J.S., Jiang Y., Geyer S., Kefauver C., Binkley P., Byrd J.C., Grever M.R., Garzon R. (2013). Pharmacokinetics and Dose Escalation of the Heat Shock Protein Inhibitor 17-Allyamino-17-Demethoxygeldanamycin in Combination with Bortezomib in Relapsed or Refractory Acute Myeloid Leukemia. Leuk. Lymphoma.

[245] Vaishampayan U.N., Burger A.M., Sausville E.A., Heilbrun L.K., Li J., Horiba M.N., Egorin M.J., Ivy P., Pacey S., LoRusso P.M. (2010). Safety, Efficacy, Pharmacokinetics, and Pharmacodynamics of the Combination of Sorafenib and Tanespimycin. Clin. Cancer Res..

[246] Schenk E., Hendrickson A.E.W., Northfelt D., Toft D.O., Ames M.M., Menefee M., Satele D., Qin R., Erlichman C. (2013). Phase I Study of Tanespimycin in Combination with Bortezomib in Patients with Advanced Solid Malignancies. Investig. New Drugs.

[247] Richardson P.G., Badros A.Z., Jagannath S., Tarantolo S., Wolf J.L., Albitar M., Berman D., Messina M., Anderson K.C. (2010). Tanespimycin with Bortezomib: Activity in Relapsed/Refractory Patients with Multiple Myeloma. Br. J. Haematol..

[248] Yust-Katz S., Liu D., Yuan Y., Liu V., Kang S., Groves M., Puduvalli V., Levin V., Conrad C., Colman H. (2013). Phase 1/1b Study of Lonafarnib and Temozolomide in Patients with Recurrent or Temozolomide Refractory Glioblastoma. Cancer.

[249] Kerklaan B.M., Diéras V., Le Tourneau C., Mergui-Roelvink M., Huitema A.D.R., Rosing H., Beijnen J.H., Marreaud S., Govaerts A.-S., Piccart-Gebhart M.J. (2013). Phase I Study of Lonafarnib (SCH66336) in Combination with Trastuzumab plus Paclitaxel in Her2/Neu Overexpressing Breast Cancer: EORTC Study 16023. Cancer Chemother. Pharmacol..

[250] Wong N.S., Meadows K.L., Rosen L.S., Adjei A.A., Kaufmann S.H., Morse M.A., Petros W.P., Zhu Y., Statkevich P., Cutler D.L. (2011). A Phase I Multicenter Study of Continuous Oral Administration of Lonafarnib (SCH 66336) and Intravenous Gemcitabine in Patients with Advanced Cancer. Cancer Investig..

[251] Kim E.S., Kies M.S., Fossella F.V., Glisson B.S., Zaknoen S., Statkevich P., Munden R.F., Summey C., Pisters K.M.W., Papadimitrakopoulou V. (2005). Phase II Study of the Farnesyltransferase Inhibitor Lonafarnib with Paclitaxel in Patients with Taxane-refractory/Resistant Nonsmall Cell Lung Carcinoma. Cancer.

[252] Hanrahan E.O., Kies M.S., Glisson B.S., Khuri F.R., Feng L., Tran H.T., Ginsberg L.E., Truong M.T., Hong W.K., Kim E.S. (2009). A Phase II Study of Lonafarnib (SCH66336) in Patients with Chemorefractory, Advanced Squamous Cell Carcinoma of the Head and Neck. Am J Clin Oncol.

[253] Shafer D., Kagan A.B., Rudek M.A., Kmieciak M., Tombes M.B., Shrader E., Bandyopadhyay D., Hudson D., Sankala H., Weir C. (2023). Phase 1 Study of Belinostat and Adavosertib in Patients with Relapsed or Refractory Myeloid Malignancies. Cancer Chemother. Pharmacol..

[254] Luu T., Frankel P., Beumer J.H., Lim D., Cristea M., Appleman L.J., Lenz H.J., Gandara D.R., Kiesel B.F., Piekarz R.L. (2019). Phase I Trial of Belinostat in Combination with 13-Cis-Retinoic Acid in Advanced Solid Tumor Malignancies: A California Cancer Consortium NCI/CTEP Sponsored Trial. Cancer Chemother. Pharmacol..

[255] O’Connor O.A., Horwitz S., Masszi T., Van Hoof A., Brown P., Doorduijn J., Hess G., Jurczak W., Knoblauch P., Chawla S. (2015). Belinostat in Patients with Relapsed or Refractory Peripheral T-Cell Lymphoma: Results of the Pivotal Phase II BELIEF (CLN-19) Study. J. Clin. Oncol..

[256] Campbell P., Thomas C.M. (2017). Belinostat for the Treatment of Relapsed or Refractory Peripheral T-Cell Lymphoma. Journal of Oncology Pharm. Pract..

[257] Maher K.R., Shafer D., Schaar D., Bandyopadhyay D., Deng X., Wright J., Piekarz R., Rudek M.A., Harvey R.D., Grant S. (2025). A Phase I Study of MLN4924 and Belinostat in Relapsed/Refractory Acute Myeloid Leukemia or Myelodysplastic Syndrome. Cancer Chemother. Pharmacol..

[258] NIH (2012). LiverTox: Clinical and Research Information on Drug-Induced Liver Injury [Internet].

[259] Xu K., Ramesh K., Huang V., Gurbani S.S., Cordova J.S., Schreibmann E., Weinberg B.D., Sengupta S., Voloschin A.D., Holdhoff M. (2022). Final Report on Clinical Outcomes and Tumor Recurrence Patterns of a Pilot Study Assessing Efficacy of Belinostat (PXD-101) with Chemoradiation for Newly Diagnosed Glioblastoma. Tomography.

[260] Herbaux C., Kornauth C., Poulain S., Chong S.J.F., Collins M.C., Valentin R., Hackett L., Tournilhac O., Lemonnier F., Dupuis J. (2021). BH3 Profiling Identifies Ruxolitinib as a Promising Partner for Venetoclax to Treat T-Cell Prolymphocytic Leukemia. Blood.

[261] Zong X., Yang Z., Zhou J., Jin Z., Wu D. (2024). Clinical Trial: Chidamide plus CHOP Improve the Survival of Newly Diagnosed Angioimmunoblastic T-Cell Lymphoma. Front. Immunol..

[262] Zou Q., Zhang Y., Zhou H., Lai Y., Cao Y., Li Z., Su N., Li W., Huang H., Liu P. (2025). Chidamide, a Histone Deacetylase Inhibitor, Combined with R-GemOx in Relapsed/Refractory Diffuse Large B-Cell Lymphoma (TRUST): A Multicenter, Single-Arm, Phase 2 Trial. Cancer Med..

[263] Chen G., Xue K., Zhang Q., Xia Z., Jin J., Li R., Liu Y., Lv F., Hong X., Li X. (2024). Chidamide plus R-GDP for Relapsed/Refractory Diffuse Large B-cell Lymphoma in Patients Ineligible for Autologous Transplantation: A Prospective, Single-arm, Phase II Study. Cancer Med..

[264] Wang W., Zhang W., Su L., Liu L., Gao Y., Wang Q., Su H., Song Y., Zhang H., Shen J. (2024). Efficacy of Chidamide Maintenance Therapy versus Autologous Stem Cell Transplantation versus Observation as a Post-Remission Choice in the Survival of Adult Patients with Peripheral T-Cell Lymphoma: Post Hoc Analysis of a Prospective, Multicenter, Phase 2 Study in China. Ann. Hematol..

[265] Liang J., Wang L., Wang X., Cui G., Zhou J., Xing T., Du K., Xu J., Wang L., Liang R. (2024). Chidamide plus Prednisone, Cyclophosphamide, and Thalidomide for Relapsed or Refractory Peripheral T-Cell Lymphoma: A Multicenter Phase II Trial. Chin. Med. J..

[266] Gao Y., He H., Li X., Zhang L., Xu W., Feng R., Li W., Xiao Y., Liu X., Chen Y. (2024). Sintilimab (Anti-PD-1 Antibody) plus Chidamide (Histone Deacetylase Inhibitor) in Relapsed or Refractory Extranodal Natural Killer T-Cell Lymphoma (SCENT): A Phase Ib/II Study. Signal Transduct. Target. Ther..

[267] Hu J., Wang J., Wang Z. (2024). The Efficacy and Safety of Chidamide in Combination with Etoposide and Glucocorticoids for the Treatment of Hemophagocytic Lymphohistiocytosis in Adult Patients: An Open-Label, Single-Center Study. Front. Immunol..

[268] Falchook G.S., Wheler J.J., Naing A., Jackson E.F., Janku F., Hong D., Ng C.S., Tannir N.M., Lawhorn K.N., Huang M. (2014). Targeting Hypoxia-Inducible Factor-1α (HIF-1α) in Combination with Antiangiogenic Therapy: A Phase I Trial of Bortezomib plus Bevacizumab. Oncotarget.

[269] Choi Y.S., Shim J., Kang K.-W., Yoon S.E., Hong J.S., Lim S.N., Yhim H.-Y., Kwon J.H., Lee G.-W., Yang D.-H. (2025). Assessing the Efficacy of Bortezomib and Dexamethasone for Induction and Maintenance Therapy in Relapsed/Refractory Cutaneous T-Cell Lymphoma: A Phase II CISL1701/BIC Study. Cancer Res. Treat..

[270] Zeng T., Jiang T., Yang G., Cheng Z., Lou C., Wei W., Tao C., Hu S., Wang H., Cui X. (2024). Bortezomib in Previously Treated Phosphatase and Tension Homology-deficient Patients with Advanced Intrahepatic Cholangiocarcinoma: An Open-label, Prospective and Single-centre Phase II Trial. Clin. Transl. Med..

[271] Fischer L., Jiang L., Dürig J., Schmidt C., Stilgenbauer S., Bouabdallah K., Solal-Celigny P., Scholz C.W., Feugier P., de Wit M. (2024). The Addition of Bortezomib to Rituximab, High-Dose Cytarabine and Dexamethasone in Relapsed or Refractory Mantle Cell Lymphoma—A Randomized, Open-Label Phase III Trial of the European Mantle Cell Lymphoma Network. Leukemia.

[272] Aubrey B.J., Blonquist T., McMasters M., Hobbs G., McAfee S., Rosenblatt J., Amrein P., Connolly C., Ramos A., Logan E. (2025). A Phase I Clinical Trial of Lenalidomide Combined with Bortezomib for Acute Myeloid Leukemia or Myelodysplastic Syndrome Relapsing after Allogeneic Stem Cell Transplantation. Leuk. Res..

[273] Gerber D.E., Boothman D.A., Fattah F.J., Dong Y., Zhu H., Skelton R.A., Priddy L.L., Vo P., Dowell J.E., Sarode V. (2015). Phase 1 Study of Romidepsin plus Erlotinib in Advanced Non-Small Cell Lung Cancer. Lung Cancer.

[274] Chiappella A., Dodero A., Evangelista A., Re A., Orsucci L., Usai S.V., Castellino C., Stefoni V., Pinto A., Zanni M. (2023). Romidepsin-CHOEP Followed by High-Dose Chemotherapy and Stem-Cell Transplantation in Untreated Peripheral T-Cell Lymphoma: Results of the PTCL13 Phase Ib/II Study. Leukemia.

[275] Bachy E., Camus V., Thieblemont C., Sibon D., Casasnovas R.-O., Ysebaert L., Damaj G., Guidez S., Pica G.M., Kim W.S. (2022). Romidepsin Plus CHOP Versus CHOP in Patients with Previously Untreated Peripheral T-Cell Lymphoma: Results of the Ro-CHOP Phase III Study (Conducted by LYSA). J. Clin. Oncol..

